# Microbes follow Humboldt: temperature drives plant and soil microbial diversity patterns from the Amazon to the Andes

**DOI:** 10.1002/ecy.2482

**Published:** 2018-10-26

**Authors:** Andrew T. Nottingham, Noah Fierer, Benjamin L. Turner, Jeanette Whitaker, Nick J. Ostle, Niall P. McNamara, Richard D. Bardgett, Jonathan W. Leff, Norma Salinas, Miles R. Silman, Loeske E. B. Kruuk, Patrick Meir

**Affiliations:** ^1^ School of Geosciences University of Edinburgh Crew Building, Kings Buildings Edinburgh EH9 3FF United Kingdom; ^2^ Smithsonian Tropical Research Institute 0843‐03092 Balboa Ancon Panama; ^3^ Department of Ecology and Evolutionary Biology Cooperative Institute for Research in Environmental Sciences University of Colorado Boulder Colorado USA; ^4^ Centre for Ecology & Hydrology Lancaster Environment Centre Lancaster LA1 4AP United Kingdom; ^5^ Lancaster Environment Centre Lancaster University Library Avenue Lancaster LA1 4YQ United Kingdom; ^6^ School of Earth and Environmental Sciences The University of Manchester Michael Smith Building, Oxford Road Manchester M13 9PT United Kingdom; ^7^ Seccion Química Pontificia Universidad Católica del Peru Av. Universitaria 1801, San Miguel Lima 32 Peru; ^8^ Department of Biology Wake Forest University Winston‐Salem NC 27109 USA; ^9^ Research School of Biology Australian National University Canberra Australian Capital Territory 2601 Australia

**Keywords:** biogeography, elevation gradient, microbial ecology, Peru, phylogenetic diversity, plant ecology, tropical forests

## Abstract

More than 200 years ago, Alexander von Humboldt reported that tropical plant species richness decreased with increasing elevation and decreasing temperature. Surprisingly, coordinated patterns in plant, bacterial, and fungal diversity on tropical mountains have not yet been observed, despite the central role of soil microorganisms in terrestrial biogeochemistry and ecology. We studied an Andean transect traversing 3.5 km in elevation to test whether the species diversity and composition of tropical forest plants, soil bacteria, and fungi follow similar biogeographical patterns with shared environmental drivers. We found coordinated changes with elevation in all three groups: species richness declined as elevation increased, and the compositional dissimilarity among communities increased with increased separation in elevation, although changes in plant diversity were larger than in bacteria and fungi. Temperature was the dominant driver of these diversity gradients, with weak influences of edaphic properties, including soil pH. The gradients in microbial diversity were strongly correlated with the activities of enzymes involved in organic matter cycling, and were accompanied by a transition in microbial traits towards slower‐growing, oligotrophic taxa at higher elevations. We provide the first evidence of coordinated temperature‐driven patterns in the diversity and distribution of three major biotic groups in tropical ecosystems: soil bacteria, fungi, and plants. These findings suggest that interrelated and fundamental patterns of plant and microbial communities with shared environmental drivers occur across landscape scales. These patterns are revealed where soil pH is relatively constant, and have implications for tropical forest communities under future climate change.

## Introduction

Climate regulates plant community composition and diversity. This observation is exemplified by the existence of changes in plant species diversity and community structure with elevation along mountainsides, first reported in a classical 19^th^ century study of the tropical Andes by the naturalist Alexander von Humboldt (von Humboldt and Bonpland 1805). However, it has remained unclear whether soil bacteria and fungi, key drivers of terrestrial biogeochemical cycling, follow similar biogeographical patterns determined by the same climatic drivers. Microbes are the most diverse and abundant organisms on Earth (Whitman et al. 1998) and perform vital metabolic functions including the decomposition of organic matter, recycling of nutrients, and formation of root symbioses, all of which can affect the productivity and diversity of plants (Bardgett and van der Putten 2014). Given their small size, abundance and short life cycles relative to plants and animals, microorganisms were long‐assumed to be cosmopolitan in their distributions (Baas Becking 1934). Recent work has challenged this paradigm, highlighting the importance of environmental filtering, historical events, stochastic speciation, and dispersal processes in shaping microbial biogeography (Fierer and Jackson 2006, Martiny et al. 2006, Tedersoo et al. 2014). Relationships between plants and soil microorganisms are now starting to be revealed (Tedersoo et al. 2014, Barberan et al. 2015, Prober et al. 2015, Zhou et al. 2016), but fundamental questions concerning their relationships over landscape‐scale gradients remain open, especially for tropical forests. The high productivity and species richness of tropical rainforests (Pianka 1966, Beer et al. 2010) translate to a greater quantity and chemical diversity of organic matter inputs to their soils and a greater diversity of plant–microbe associations (Hattenschwiler et al. 2008, Mangan et al. 2010, Fanin et al. 2014). Together, these characteristics point towards more opportunities for associations between plant and microbial species in tropical than in temperate or high‐latitude biomes, potentially leading to stronger coordinated changes in all major biota across climatic gradients within tropical forests.

The large temperature gradients on mountains have proven invaluable for understanding how temperature influences plant diversity, community composition, and productivity (Colwell et al. 2008). Shifts in the diversity of plant and animal taxa with changes in elevation along mountainsides globally are thought to result principally from differences in energy limitation and/or niche differentiation, leading to a typically monotonic decrease or mid‐elevation peak in aboveground species richness with elevation (Rahbek 2005). Elevation gradients can also help us to understand the influence of temperature on the diversity and functional attributes of soil microbial communities and their role in soil organic matter cycling (Bryant et al. 2008, Geml 2017). However, such studies have not shown the strong elevation‐related pattern of diversity almost universally observed for plants. Studies of bacterial richness have revealed contrasting patterns, strongly influenced by multiple additional drivers, particularly the large between‐sample variations in rainfall or soil pH that have accompanied such studies (Bryant et al. 2008, Shen et al. 2013, Singh et al. 2014, Peay et al. 2017). Similarly, contrasting patterns have been found in studies of fungal richness, which have generally targeted specific groups that vary in their elevation relationship by functional type and plant‐host specificity (reviewed in Geml 2017, Kivlin et al. 2017). Any of these sources of sample variance could obscure an underlying fundamental relationship between temperature and microbial diversity.

The diversity and functional attributes of bacteria and fungi along elevation gradients in tropical forests are especially poorly resolved despite their high biodiversity. We would expect the biogeographical patterns of plants and soil microorganisms to be related, as suggested by studies that have associated microbial communities with plant leaf litter traits (Orwin et al. 2010, de Vries et al. 2012, Handa et al. 2014). The wide inter‐specific variation in leaf traits reported for tropical forests (Hattenschwiler et al. 2008, Salinas et al. 2010, van de Weg et al. 2009) therefore points towards stronger associations between plant leaf traits (e.g. chemical diversity) and soil microbial species assemblages, relative to other ecosystems. Where this question has been addressed in the tropics, a relationship between the chemical composition of leaf‐litter and the underlying microbial community composition has been demonstrated in an incubation experiment (Fanin et al. 2014), but there was no overall relationship between plant and soil microbial species diversity in a study of a single, albeit large, forest plot in Panama (Barberan et al. 2015). However, the issue has not yet been investigated at a larger biogeographical scale in the tropics. A global study of grasslands found relationships between plant, bacterial, and fungal diversity at the community level (β‐diversity), but not at the level of species richness (α‐diversity; Prober et al. 2015). Plant and fungal α‐diversity were positively related across a global latitudinal gradient (Tedersoo et al. 2014) and detailed relationships have been shown for specific groups of fungi (Geml 2017, Kivlin et al. 2017). Importantly, these biogeographical patterns have not been observed for bacteria across gradients in latitude or elevation (Bardgett and van der Putten 2014, Prober et al. 2015), possibly due to the wide variation in soil pH, which has likely confounded sampling for biogeographical patterns in bacteria (Fierer and Jackson 2006). In summary, whilst some work points towards related biogeographical patterns among plant and microbial communities (Tedersoo et al. 2014, Prober et al. 2015), the evidence is often inconclusive or partly contradictory (de Vries et al. 2012, Barberan et al. 2015), and especially so for tropical forest.

**Figure 1:**
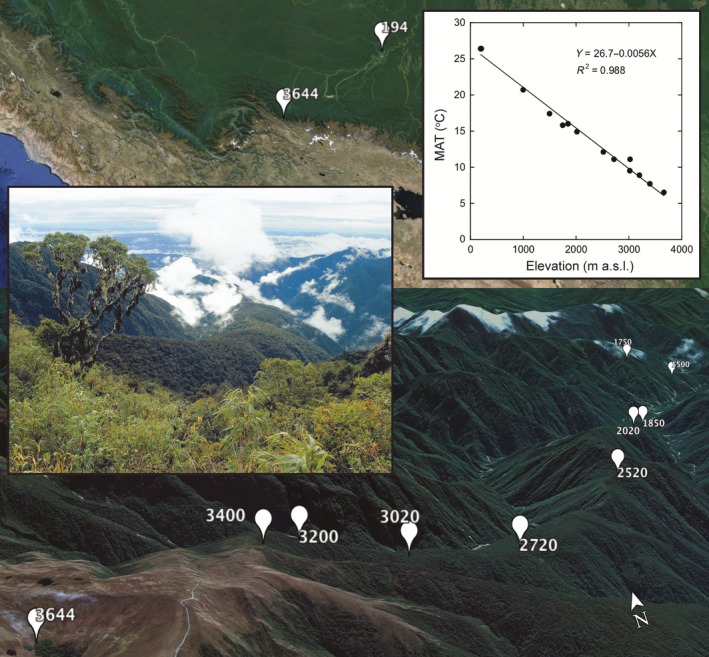
The Kosñipata elevation transect, Manu National Park, Peru. The top panel shows the highest (3,644 m above sea level [a.s.l.]) and lowest elevation (194 m a.s.l.) sites and the relationship between elevation and mean annual temperature (MAT). The bottom panel shows all sites from 3,644 to 1,500 m a.s.l. viewed facing approximately northeast from the top of the transect. The photograph shows a northeasterly view from approximately 3,500 m a.s.l. along the transect.

In this study, we used a 3.5‐km tropical elevation gradient (equivalent to a 6.5°–26.4°C mean annual temperature range) in the Peruvian Andes (Fig. 1) to ask: (1) whether related biogeographical patterns in plant, bacterial, and fungal species richness (α‐diversity) and compositional dissimilarity of communities (β‐diversity) occur across large environmental gradients; and (2) whether temperature drives these patterns where other key environmental variables are constrained. Importantly, the variation along this elevation gradient in the key environmental variables of soil pH and moisture is small (Appendix S1: Table S1), meaning that our data should be minimally affected by other principal potential environmental factors that might be expected to confound the observation of any fundamental effect of temperature on soil biota. We sampled at high density considering the logistical challenges of the environment (14 sites in total, with soil samples from two separate horizons). We determined the α‐diversity and β‐diversity for plants and soil microbes by using field surveys of 1‐ha permanent sample plots for plants and high‐throughput sequencing for soil microbes. Our analyses included a large suite of environmental and soil properties, including soil extracellular enzyme activity, to determine the environmental drivers of these patterns in plant and microbial diversity, and how the patterns were related to indices of organic matter cycling.

## Materials and Methods

### Study sites

The elevation transect under study lies on the Eastern flank of the Andes in southeastern Peru, in the upper Madre de Dios/Madeira watershed (Fig. 1; Nottingham et al. 2015a). The transect spans 3,450 m in elevation from 194 to 3,644 m above sea level (asl) and consists of 14 sites, each with a 1‐ha permanent sampling plot, all in old growth tropical forest except for one site on high elevation grassland (Fig. 1, Appendix S1: Table S1). The sites are roughly evenly distributed by elevation but not in terms of spatial separation: the transect is approximately 270 km in length, with 35 km between the upper 12 sites and 12 km between the upper nine sites. Mean annual temperature (MAT) decreases with increasing elevation across the transect (dropping from 26° to 6°C), but mean annual precipitation (MAP) does not vary consistently with elevation, ranging from 1,506 to 5,302 mm/yr among the sites (Girardin et al. 2013). Seasonality of MAP decreases with increasing elevation, with no clear dry season in the montane forest sites (Girardin et al. 2013) and approximately 4 months (June–September) with <100 mm rainfall in the lowland forest sites (Malhi et al. 2014); although there is no evidence of seasonal soil or plant moisture constraints (Zimmermann et al. 2010, van de Weg et al. 2014). The plots are situated on predominantly Paleozoic (~450 Ma) meta‐sedimentary mudstone (~80%), with plutonic intrusions (granite) underlying the sites between 1,500 and 2,020 m asl. The soils at the sites above 2,520 m are Umbrisols (Inceptisols), while the soils from 1,000 to 2,020 m are Cambisols (Inceptisols). The soils below 1,000 m, at the two lowland sites, are Haplic Allisols (Ultisols) and Haplic Cambisols (Inceptisols) (according to FAO, with USDA Soil Taxonomy in parentheses). Further descriptions of soil, climate, and floristic composition of these sites are reported elsewhere (Rapp et al. 2012, Jankowski et al. 2013, Whitaker et al. 2014).

#### Plant and soil data collection

Soil and microbial properties were determined for 14 sites (13 forest, 1 high elevation grassland). Plant diversity was determined in the 13 forest sites, resulting in 13 sites with both tree and microbial data. For the 13 forest sites, trees were measured in each 1‐ha plot, where every individual tree ≥10 cm diameter at breast height (1.3 m) was measured, tagged, and identified to species or morphospecies. Plants were censused during 2007–2012; for further details on methodology see Rapp et al. (2012). For all sites, soil samples were collected during January 2012 from five systematically distributed sampling points in the 1‐ha plots. Given that these ecosystems are largely aseasonal, with no significant intra‐annual variation in mean monthly temperature across all sites and no evidence of seasonal soil or plant moisture constraints (Zimmermann et al. 2010, van de Weg et al. 2014), the comparison of soil properties for these sites at a single time point was considered representative of patterns likely to be found throughout the year. We used composite soil samples composed of three replicates for DNA extraction because our aim, for both plants and soil microorganisms, was to characterize the overall diversity and community composition by plot (rather than to investigate the spatial variation within the plot). However, we used five spatial replicates for all other edaphic analyses, to quantify the within‐plot variation for soil properties. The five spatial replicates were sampled from outside each of the four corners, and from one central point, of the 1‐ha plot. We collected and analysed samples from both organic and mineral horizons, with the mineral horizon samples coming from the upper 10 cm of the mineral layer. Soil samples were stored for <14 d at <4°C until DNA extraction and determination of nutrient content and enzyme activities; this method has been shown to have negligible effects on these soil properties (Lauber et al. 2010, Turner and Romero 2010).

### Soil analyses: DNA sequencing, nutrients and extracellular enzyme activities

Microbial diversity was assessed using high‐throughput sequencing to characterize the variation in marker gene sequences (Fierer et al. 2012). For bacterial community composition, the 16S rRNA gene was amplified in triplicate PCR reactions using the 515f and 806r primers. For fungal community composition, the first internal transcribed spacer region (ITS1) of the rRNA gene was amplified using the ITS1‐F and ITS2 primer pair. Raw sequence data were processed using the QIIME v1.7 pipeline, where sequences were de‐multiplexed using their unique barcode specific to individual samples and assigned to phylotypes (operational taxonomic units, OTUs, at 97% similarity). Taxonomy was determined for each phylotype using the RDP classifier (Wang et al. 2007) trained on the Greengenes (McDonald et al. 2012) and UNITE (Abarenkov et al. 2010) databases for bacterial and fungal sequences (see Supplementary Information for further detail).

#### Soil characteristics

We determined the following soil variables: total carbon (C), total nitrogen (N), total phosphorus (P), organic P, resin‐extractable P (resin P), resin‐N, effective cation exchange capacity (ECEC) and exchangeable cations (Al, Ca, Fe, K, Mn, Mg, Na), soil pH, bulk density, moisture content, and activities of seven soil enzymes (Nottingham et al. 2012). We determined 31 soil and environmental variables in total (see Supplementary Information for further detail).

### Measures of α‐ and β‐diversity

We analyzed estimates of α‐ and β‐diversity for each biotic group. For plants, α‐diversity measures came from Shannon species diversity indices for each plot and therefore constituted a single measure per site (*n* = 13 sites in total, as there was no plant diversity measure from the grassland site). Bacterial and fungal α‐diversity were determined using Shannon species diversity indices based on the abundance of OTUs for soil bacteria and fungi. We determined β‐diversity (community composition) using dissimilarity matrices (Sørensen and Bray‐Curtis for plants and soil microbes, respectively). The process was repeated separately for the organic and the mineral horizons. Our data set therefore consisted of the following five measures of α‐diversity and β‐diversity for each site: plants, fungal‐organic, fungal‐mineral, bacterial‐organic, and bacterial‐mineral.

### Statistical analyses

Our main hypotheses that (1) α‐ and β‐diversity measures across biotic groups are related and (2) have shared environmental drivers, were addressed by using linear and linear mixed‐models for α‐diversity (mixed effects models) and multivariate methods for β‐diversity (permutational‐MANOVA (PERMANOVA), principal coordinates analyses (PCA), Mantel tests, and multivariate correlation models). The effects of elevation on α‐diversity were tested using a linear model for plant diversity and linear mixed‐effects models for each of the four measures of microbial diversity. Elevational differences in β‐diversity were examined using PERMANOVA, PCA, and Mantel tests. The effects of climate and edaphic variables on α‐diversity were addressed by testing for effects of seven variables on α‐diversity: MAT, MAP, pH, total C, ECEC, resin P, and between‐plot distance, using a linear model for plant diversity, and linear mixed models for each of the four measures of soil microbial diversity. We restricted linear models to include only seven variables, which were selected due to their known influences on plant and soil microbial communities. To test for covariance among model parameters, we calculated variance inflation factors (VIFs) for all parameters in the final model. To test for the effects of climate and edaphic variables on β‐diversity, we used BIO‐ENV multivariate correlation models (Clarke and Ainsworth 1993), which creates a model by step‐wise selection, determining high‐rank correlations between species dissimilarity matrices and environmental resemblance matrices. The environmental resemblance matrices can be generated from a large set of environmental variables. Detailed descriptions of these statistical tests are provided in supplementary material. All statistical analyses were performed in either R (version 3.4.1) or PRIMER (version 6.1.12; PRIMER‐E, Plymouth, UK). The combined analysis allowed us to (1) determine whether diversity patterns in plants, bacteria, and fungi are related; (2) infer the principal environmental or edaphic drivers of the observed patterns in diversity; and (3) test whether the diversity and community composition of soil microorganisms influence soil processes along a tropical elevation gradient.

## Results

### Effect of elevation on α‐diversity

**Figure 2:**
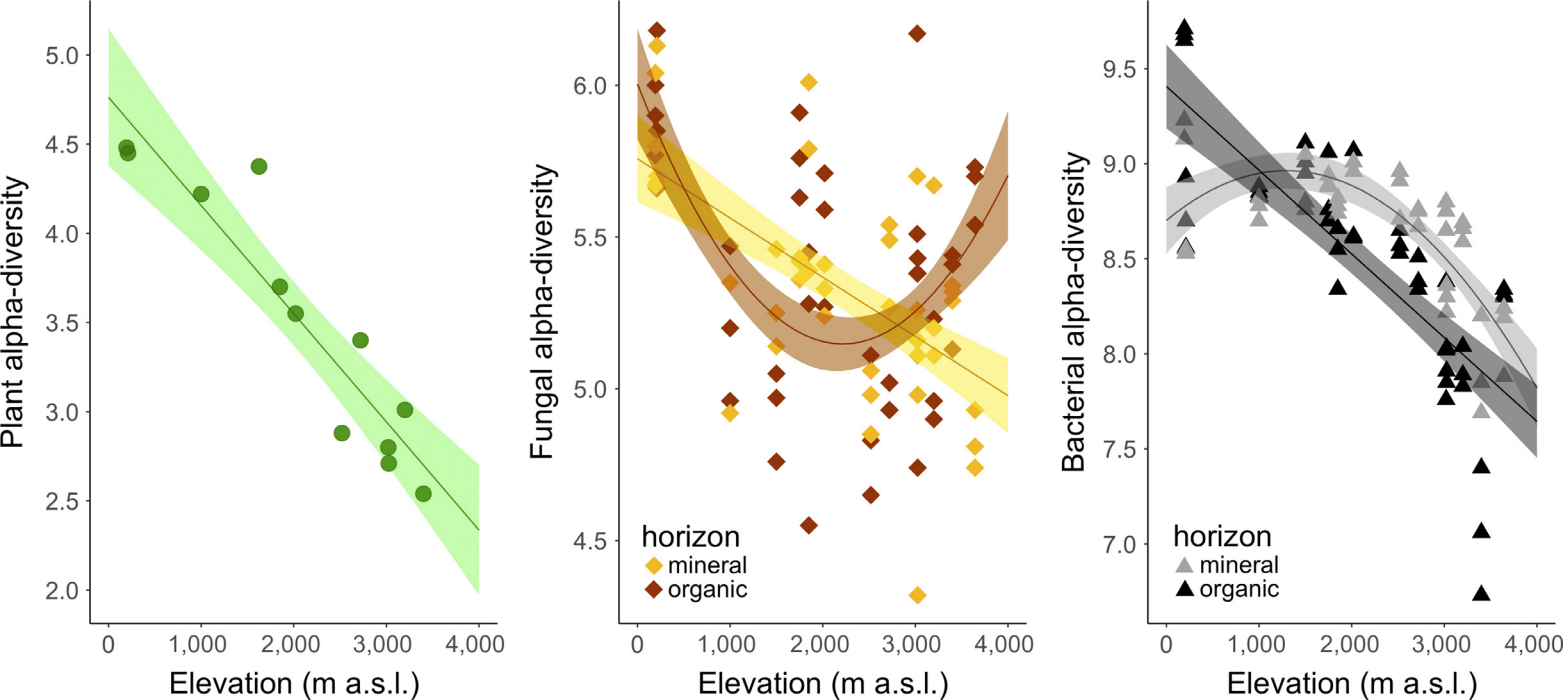
Changes in α‐diversity with elevation in plants, fungi, and bacteria. Fungi and bacteria were sampled from both the organic and the mineral soil horizon, and each site is represented by three data points. Note the different scales on the *y*‐axes. The solid lines and confidence intervals show predicted relationships and 95% confidence intervals from models equivalent to those shown in Appendix S1: Table S2 but excluding quadratic terms where they were non‐significant, i.e., for the plants, fungal‐organic, and bacterial‐mineral groups.

There were significant differences between biotic groups in their average levels of α‐diversity (linear mixed model, group effect: *F* = 1093; df = 4,165; *P *<* *0.001), which increased in the order: plants < fungi < bacteria (Fig. 2). For each group, all α‐diversity measures declined with increased elevation, except for fungal diversity in the organic horizon (Appendix S1: Table S2). For plant, fungal‐mineral and bacterial‐organic α‐diversity, the decline with elevation was best described with a linear model (Appendix S1: Table S2a, c, d). For fungal‐organic and bacterial‐mineral α‐diversity, the change was non‐linear: for fungal‐organic, α‐diversity was lowest at mid‐elevation (Fig. 2b, Appendix S1: Table S2b), whereas for bacterial‐mineral, α‐diversity only showed significant declines at the higher elevations (Fig. 2c, Appendix S1: Table S2e). There was no significant spatial autocorrelation of residuals for all models (Appendix S1: Table S2; Moran's I; Ape package in R). The effects of elevation on α‐diversity were therefore mostly negative, but there were also significant differences among groups in the exact pattern of change with elevation (combined linear mixed model, group × elevation, *F* = 23.48; df = 5,87, *P *<* *0.001; group × elevation^2^, *F* = 7.80; df = 5,86; *P *<* *0.001). Plants showed the steepest decline in α‐diversity with elevation (slope = −0.753 ± 0.094; Appendix S1: Table S2).

### Effects of climate and edaphic variables on α‐diversity

Mean annual temperature (MAT) was the dominant determinant of the patterns in α‐diversity for all biotic groups and in both soil horizons, with the exception, again, of fungal‐organic (Table 1). Plant α‐diversity increased significantly with MAT, but also increased with mean annual precipitation (MAP; Table 1a). Fungal‐organic α‐diversity was not correlated to either climatic variable, but instead declined significantly with increasing ECEC (Table 1b). For both mineral horizon measures (fungal and bacterial), the only variable with any significant effect was MAT (Table 1c, e). Bacterial‐organic α‐diversity was also positively affected by MAT, and additionally by resin‐P (Table 1d). There was no evidence for significant covariance of variables in the final models (VIFs for all parameters in final model < 2). Full models with all seven variables showed the same qualitative results (Appendix S1: Table S3); spatial separation had no significant effect. In summary, temperature (MAT) had a near‐universally significant positive effect on α‐diversity, but precipitation (MAP), ECEC and resin‐P were all also relevant for certain biotic group‐soil horizon combinations.

**Table 1:** Final models of effects of climatic and edaphic parameters on α‐diversity in the five groups

Parameter	Mean	SE	*t*	*P*	Prop. variance	Variance	*R* ^2^
(a) Plants							
(Intercept)	1.312	0.179	7.324	0			
MAT	0.108	0.009	12.29	0	0.821		
MAP	2.393 × 10^−4^	0.438 × 10^−4^	5.461	0	0.119		
*R* ^2^	0.940						
(b) Fungal organic							
(Intercept)	5.904	0.158	37.449	0			
ECEC	−0.012	0.003	−3.823	0.002	0.385		
Random effects							
Site						0.040	
Residual						0.066	
Marginal							0.385
Conditional							0.619
(c) Fungal mineral							
(Intercept)	4.834	0.16	30.201	0			
MAT	0.034	0.01	3.382	0.004	0.342		
Random effects							
Site						0.037	
Residual						0.049	
Marginal							0.342
Conditional							0.627
(d) Bacterial organic							
(Intercept)	8.056	0.247	32.64	0			
MAT	0.048	0.013	3.645	0.003	0.575		
resinP	−2.33 × 10^−3^	−0.58 × 10^−3^	−3.972	0.001	0.199		
Random effects							
Site						0.057	
Residual						0.029	
Marginal							0.774
Conditional							0.922
(e) Bacterial mineral							
(Intercept)	8.213	0.19	43.2	0			
MAT	0.031	0.012	2.568	0.022	0.294		
Random effects							
Site						0.071	
Residual						0.013	
Marginal							0.294
Conditional							0.889

*Notes*

Final models after removal of all non‐significant variables: linear model for plants (*n* = 13) and linear mixed models for fungi/bacteria (with site as random effect; *n* = 42). MAT, mean annual temperature; MAP, mean annual precipitation; ECEC, cation exchange capacity; resinP, resin‐extractable P (for full models with all six variables for each measure of α‐diversity, see Appendix S1: Table S3). Prop. variance gives the proportion of variance explained by each fixed effect; marginal *R* ^2^ is that explained by all the fixed effects together; conditional *R* ^2^ is that explained by both fixed and random effects (see Materials and Methods).

### Correlations between α‐diversity of different groups

**Figure 3:**
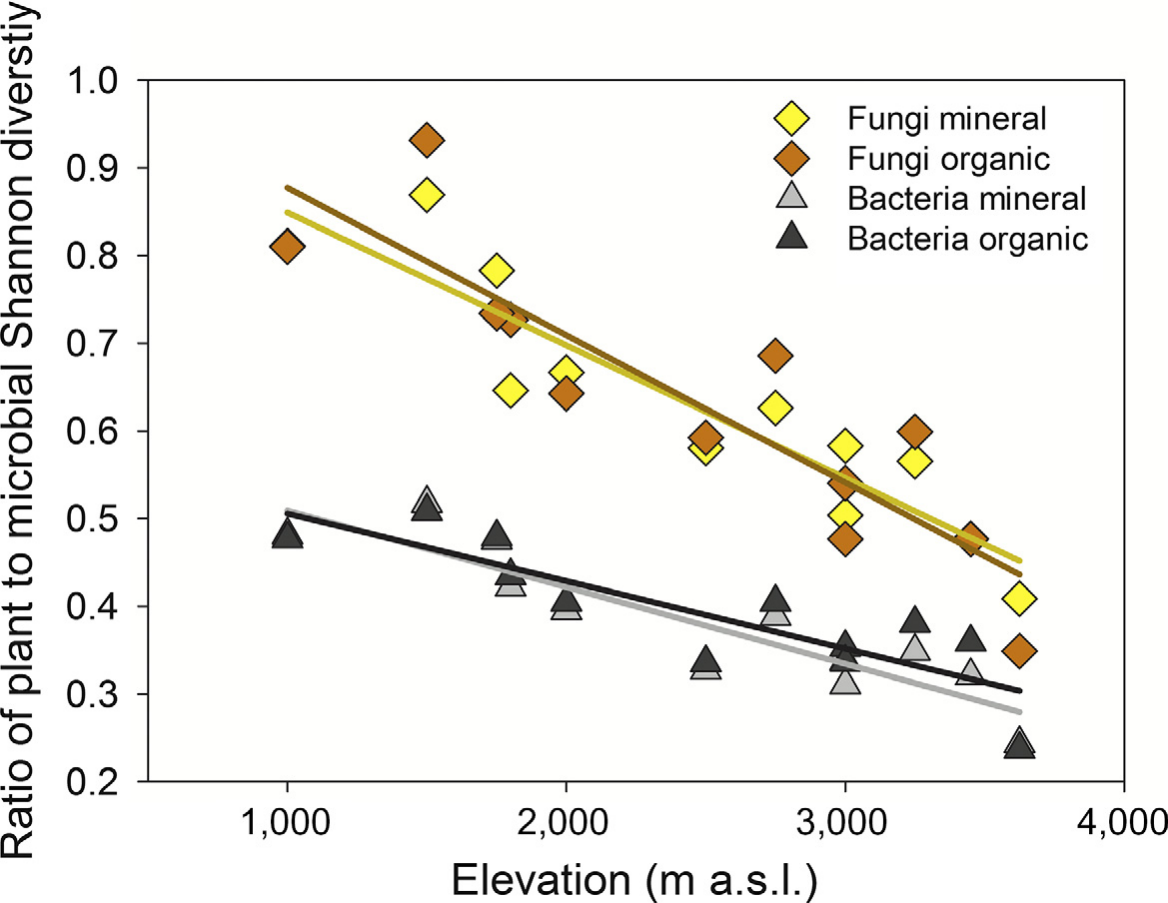
The relationships between the ratios of plant to bacterial and plant to fungal α‐diversity and elevation in organic and mineral soil horizons. Regression lines are shown with for plants:bacteria and plants:fungi against elevation in both mineral and organic horizons. The stronger coupling of plant and bacterial diversity (Spearman's correlation: ρ = 0.83, 0.57; organic and mineral horizons, respectively) compared to plant and fungal diversity (ρ = 0.60, 0.39), was further reflected in a greater decline with elevation for the species richness ratio of plants to fungi (average slope of 1.02) compared to plants to bacteria (average slope of 0.59).

Plant α‐diversity was most strongly positively correlated with that of bacterial α‐diversity, especially in the organic horizon (*r* = 0.83; Appendix S1: Table S4). The coupling of plant and bacterial α‐diversity also appeared to be more conserved than for plant and fungal α‐diversity: the ratio in the Shannon diversity index for plants:bacteria varied with elevation by less than half that for plants:fungi, in both mineral and organic horizons (Fig. 3). There were also strong positive correlations between α‐diversity for the two soil horizons for bacteria (*r* = 0.81), but not for fungi (*r* = 0.30). Overall, the fungal organic α‐diversity showed the weakest coupling to any of the other measures (Appendix S1: Table S4).

### Elevation patterns in β‐diversity of different groups

**Figure 4:**
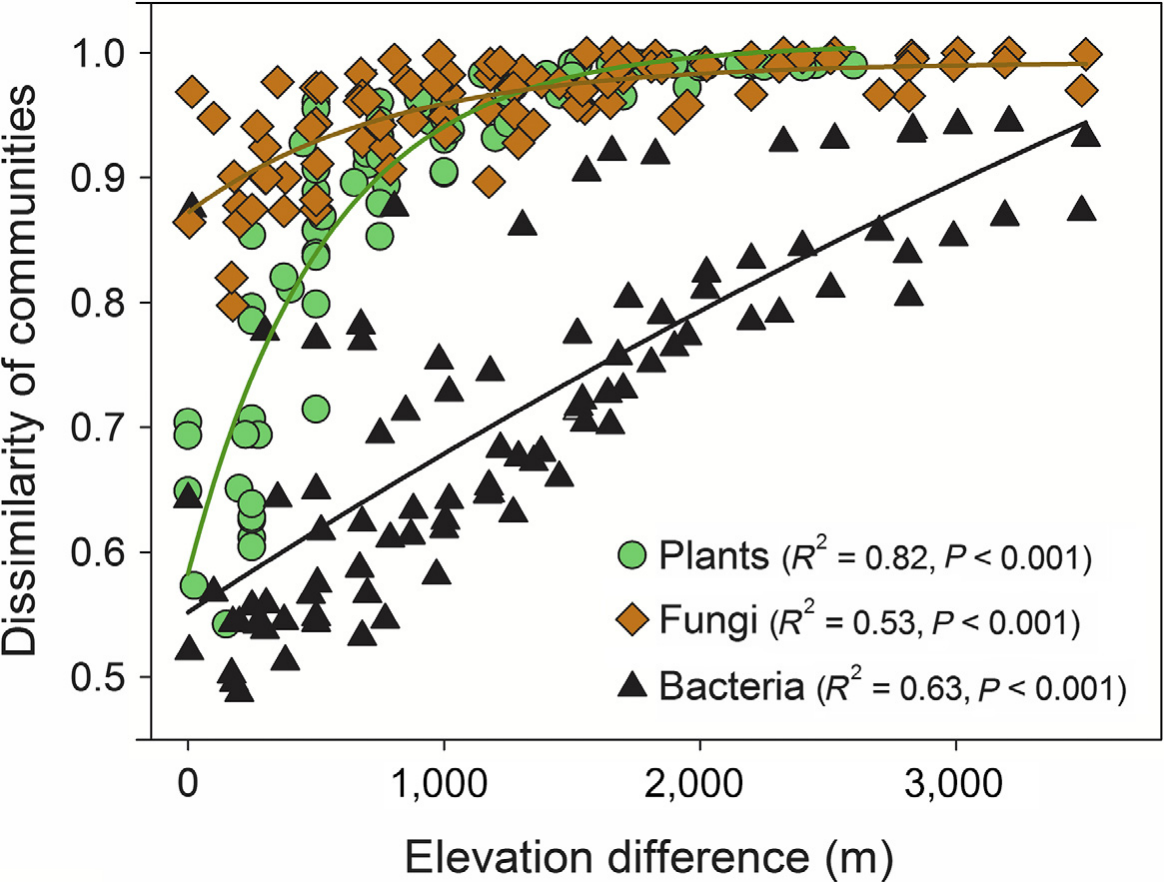
The relationship between ß‐diversity of plants, bacteria and fungi (dissimilarity of communities) with elevation difference. ß‐diversity for all groups differed with elevation (plants: *P *<* *0.001, *F* = 79.2, df = 19; bacteria: *P *<* *0.001. *F* = 4.5, df = 69; fungi: *P *<* *0.001, *F* = 3.3, df = 83; by PERMANOVA). Soil microbial data are shown for organic soil horizons (there were consistent patterns in mineral horizons). The overall decline with increased elevation indicates increased dissimilarity in ß‐diversity between sites with greater difference in elevation. Elevational declines were fitted with exponential models [*y* = *a*[1 − exp(−*bx*)]; with parameter estimates for bacteria (*a* = 1.27, 0.001), fungi (*a* = 0.12, *b* = 0.0013) and plants (*a* = 0.424, *b* = 0.0019)].

The composition of plant, bacterial, and fungal communities differed with elevation (differences in β‐diversity; all comparisons by PERMANOVA; *P *<* *0.001; Fig. 4) and was characterized by exponential relationships whereby community compositional dissimilarity tended towards a maximum (dissimilarity = 1) with increased elevational separation (Fig. 4). Fungi exhibited the largest compositional dissimilarities of communities with elevation, followed by plant and then bacteria communities. The β‐diversity of bacteria and fungi also differed between organic and mineral horizons (Appendix S1: Fig. S2), although fungi differed to a smaller extent than bacteria (all comparisons by PERMANOVA; *P *<* *0.001).

The β‐diversity of plant communities was high across the gradient and there was a steep increase in dissimilarity with increased elevation difference (Fig. 4). This high β‐diversity was likely driven by high species turnover along narrow local elevational ranges, with a species occurring on average in only 1.7 plots (Jankowski et al. 2013). This species‐level turnover is underlain by deeper phylogenetic shifts, with turnover in the dominant families and orders in the tree community along the gradient. At low elevations, tree communities are dominated by the families Euphorbiaceae, Urticaceae, Fabaceae, Moraceae, and Arecaceae, shifting to communities dominated by Cunoniaceae, Melastomataceae, Clethraceae, Clusiaceae, and Symplocaceae at high elevations; with corresponding ordinal changes being Malphigiales, Rosales, and Fabales at low elevations shifting to Ericales, Myrtales, and Oxalidales at high elevations.

The differences in soil microbial β‐diversity with elevation were reflected by shifts in dominant phyla (Appendix S1: Fig. S3). For bacteria, increased elevation was associated with an increased dominance of Acidobacteria and Betaproteobacteria, and decreased dominance of Actinobacteria and Deltaproteobacteria; the patterns occurred in both horizons, although mineral horizons contained a greater proportion of Acidobacteria (Appendix S1: Fig. S3a). For fungi, increased elevation was associated with increased dominance of Ascomycota (Archaeorhizomycetes, Leotiomycetes), Basidiomycota (Microbotryomycetes), and decreased dominance of other Ascomycota (Sodariomycetes, Dothideomycetes, Eurotiomycetes) and Glomeromycota (Appendix S1: Fig. S3b, Table S5). The β‐diversity patterns observed for bacteria and fungi were correlated with those observed for plants (Fig. 4). Patterns in ß‐diversity were correlated between plants and bacteria (organic horizon ρ = 0.81; mineral horizon ρ = 0.88) and plants and fungi (organic horizon ρ = 0.67; mineral horizon ρ = 0.79; by Mantel tests; *P *<* *0.001 for all comparisons). Thus, plants and several major taxonomic groups of both bacteria and fungi showed clear and correlated changes in composition with elevation.

### Effects of climate and edaphic variables on β‐diversity

As with α‐diversity, MAT was the strongest correlate of patterns in β‐diversity. MAT was the most significant parameter in multivariate models (Bioenv; vegan package, R) for β‐diversity of plants, bacteria in both organic and mineral horizons, and fungi in mineral horizons (Table 2). There were additional correlations between the β‐diversity of bacteria and fungi, and dissimilarity matrices of organic nutrient concentrations and their ratios; these were stronger in the organic compared to mineral horizons (Appendix S1: Fig. S4). Nutrients other than N and P were also correlated with β‐diversity, including K for plants and Na for bacteria (Table 2). Soil pH affected bacterial β‐diversity, but not fungal β‐diversity (Table 2).

**Table 2:** The effects of environmental and edaphic variables on plant, bacterial and fungal β‐diversity, determined by multivariate correlation models

		Organic horizon		Mineral horizon	
Variable	Plants	Bacteria	Fungi	Bacteria	Fungi
MAT	(0.91)***	(0.77)***	(0.67)*	(0.88)***	(0.59)***
Soil pH	ns	(0.57)*	ns	(0.47)**	ns
Total C:N	ns	(0.68)**	ns	ns	(0.38)**
Total C:P	ns	ns	(0.70)**	ns	ns
Na	ns	ns	ns	(0.22)*	ns
K	(0.44)**	ns	ns	ns	ns
*N*‐acetyl β‐glucosaminidase	ns	ns	(0.74)***	ns	ns
Complete model	0.93	0.88	0.80	0.91	0.65

*Notes*

The final models were determined by step‐wise selection to determine which resemblances matrices for 47 initial predictor variables best describe community composition dissimilarity matrices. Significance of individual parameters in each model was determined by Mantel tests between β‐diversity and the specific variable, shown in parentheses. Values are correlation coefficients.

***P <* *0.001; **P <* *0.01; *P <* *0.05; ns, not significant.

### Soil β‐diversity and function

The activities of seven soil enzymes decreased with increased elevation but at different rates, and independently of differences in ambient temperature (Appendix S1: Fig. S5). These patterns reflected responses in the microbial community to shifts in substrate availability in the soil. For example, relative microbial investment into different enzymes shifted with increased elevation, from enzymes that degrade P‐ to N‐ containing organic compounds (Nottingham et al. 2015b). Strong relationships between the differential activity of these seven enzymes and differences in β‐diversity were found for bacteria (ρ = 0.75) and fungi (ρ = 0.74) in organic horizons (Appendix S1: Fig. S6; by Mantel tests*; P *<* *0.001 for all comparisons).

## Discussion

Overall, our results demonstrate a fundamental role for environment, principally temperature, in coordinating the diversity and community composition of plants, soil bacteria, and fungi along an extensive 3.5‐km elevation gradient in tropical forest. For all three biotic groups, species richness (α‐diversity) declined as elevation increased, and the compositional dissimilarity of communities (β‐diversity) increased with increased elevation difference between communities, although the changes in plant α‐diversity were larger than in bacteria and fungi (Figs. 2, 3). While environmental filtering at large geographic scales has been suggested to shape community composition for plants, soil bacteria, and fungi independently (Tedersoo et al. 2014, Prober et al. 2015), this has not been reported before for both α‐diversity and β‐diversity and across all three biotic groups together. Fundamentally, temperature, and to a much lesser extent rainfall and edaphic properties, were strongly associated in our data with variation in plant, bacterial, and fungal α‐diversity (from linear models; Table 1) and β‐diversity (from multivariate models; Table 2).

Plant community shifts along elevational gradients have long been thought to correspond to temperature changes (von Humboldt and Bonpland 1805), and comprise a classic biogeographic pattern in both tropical and temperate zones (Whittaker 1956, Gentry 1988). Our study confirms a dominant role for temperature in driving both the steep decrease in plant α‐diversity with increasing elevation (Fig. 2) and the high β‐diversity across the gradient (Table 2), which is, in turn, due to high turnover of species with narrow elevation ranges (Jankowski et al. 2013). Patterns in plant species composition and richness on tropical mountains are thought to be driven mainly by the effects of geographically narrow temperature ranges on niche separation (by directly affecting metabolism and indirectly affecting resource availability), further constrained by land area, lithology, fertility, and disturbance history (Janzen 1967, Colwell et al. 2008, Prada et al. 2017). Although the high landslide activity and soil erosion in the humid Eastern Andean Cordillera (Clark et al. 2013) may be factors in constraining overall diversity at higher elevations in this region, our study identifies a central underlying role for temperature. Indeed, temperature has previously been shown to be a major determinant of tree community composition across this transect (Rapp et al. 2012), and up‐slope movement of tree species’ lower‐range limits has been observed under recent climatic warming (Feeley et al. 2011, Duque et al. 2015).

While temperature was the main determinant of high plant β‐diversity across the gradient (Table 2), the highest plant β‐diversity occurred across the mid‐elevation sites corresponding to the zone at which persistent cloud immersion begins (Fyllas et al. 2017). This suggests an additional influence of moisture and light in determining compositional changes at the mid‐elevations, perhaps through impacts on productivity (Goldsmith et al. 2013, Fyllas et al. 2017). The mid‐elevation zone is also where there is a transition in soil nutrient availability from N to P (Nottingham et al. 2015b), which may promote plant β‐diversity according to different nutrient‐use strategies (Condit et al. 2013). Although the pattern of shifts in plant β‐diversity along elevation gradients is well described, and there is intensive work on plant functional traits on this transect (Asner et al. 2014, Enquist et al. 2017, Fyllas et al. 2017, van de Weg et al. 2009, van de Weg et al. 2012, van de Weg et al. 2014), the mechanistic basis of the generation of this pattern is not yet well resolved (Silman 2014), but will ultimately inform observed on‐going and past shifts in climate and plant species range shifts (Bush et al. 2004, Feeley et al. 2011).

As with the plant diversity metrics, soil microbial α‐diversity decreased with increasing elevation (Fig. 2), β‐diversity was high across the gradient (Fig. 3), and temperature was the dominant driver of these patterns (Table 2); although there were differences in the strength of diversity gradients and in the secondary drivers of these patterns. The role of temperature in determining microbial β‐diversity is illustrated by shifts in the relative abundance of specific taxonomic groups. For example, there was an increased relative abundance of Acidobacteria and the fungi Archaerhizomycetes with increased elevation, but a decreased relative abundance of Actinobacteria and Alphaproteobacteria (Appendix S1: Fig. S3). These major taxonomic groups have been associated with oligotrophic (Acidobacteria, Archaerhizomycetes) and copiotrophic (Actinobacteria, Alphaproteobacteria) life history strategies, respectively (Fierer et al. 2007, Rosling et al. 2011), which is consistent with evidence for increased energy limitation at higher, cooler elevations (e.g. decreased decomposition rates) (Bruijnzeel et al. 2011, Nottingham et al. 2015a), favoring slower growth. The high relative abundance of the Ascomycota, Archaerhizomycetes at higher elevations (Appendix S1: Fig. S3) is of particular interest because this class of fungus was discovered only recently and their global distribution is poorly understood, partly because many previous analyses failed to identify them due to amplification biases (Rosling et al. 2011). They were recently identified in a range of global biomes, but generally represented <1% of relative abundance (Tedersoo et al. 2014), which contrasts with their high relative abundance in our upper montane forest sites (26%; Appendix S1: Fig. S1B). They are understood to be typically oligotrophic and root‐associated fungi (Choma et al. 2016), colonizing typical ectomycorrhizal (EM) fungal habitats (Rosling et al. 2011). This important class of fungi, which until very recently was unknown, is a major component of the fungal biomass in these tropical montane forests.

Diversity gradients were steeper for plants compared to microorganisms (Figs. 2, 3), which is consistent with the widespread view that microorganisms are more diverse and more cosmopolitan in their distributions than plants (Martiny et al. 2006). Analogous plant/microbial diversity relationships have been shown along latitudinal gradients where plant/microbial species richness ratios decrease with distance from the equator (Tedersoo et al. 2014, Zhou et al. 2016). Our data indicate a stronger coupling between the α‐diversity of plants and bacteria compared to fungi (Fig. 3, Appendix S1: Table S4), but a stronger coupling between the β‐diversity of plants and fungi than for bacteria (Fig. 4). The stronger coupling between the α‐diversity of plants and bacteria may indicate stronger biotic interactions between plants and bacteria compared to plants and fungi, although controlled experiments are required to understand the nature of these interactions (for example, the interaction between litter chemistry and microbial communities). The stronger coupling between the β‐diversity of plants and fungi can be explained by coordinated shifts in the presence of obligate plant hosts among sites for symbiotic fungi (Geml 2017); which can also explain the absence of a clear elevation pattern in fungal α‐diversity in organic soil horizons (Fig. 4). Consistent with this idea, correlations between plant and fungal β‐diversity alongside high variation in α‐diversity patterns among specific fungal phyla (especially those that form plant associations) have been observed across elevation gradients in a range of ecosystems (Geml et al. 2014, 2017, Merckx et al. 2015, Looby et al. 2016, Geml 2017). For example, along other elevation gradients, the presence of EM and endophytic fungal hosts explained correlations in plant and fungal β‐diversity (Geml et al. 2014) and opposing α‐diversity patterns have been observed for arbuscular mycorrhizal (AM) and EM fungi (Geml et al. 2017, Kivlin et al. 2017). The lack of a clear relationship in our data between temperature and α‐diversity for the distinct fungal communities in organic horizons (Fig. 3, Appendix S1: Fig. S2) may therefore reflect a stronger signal of plant–host associations on total fungal α‐diversity.

In addition to the main effect of MAT, there was a secondary role for other environmental and edaphic properties in shaping these diversity patterns (Tables 1, 2; Appendix S1: Table S3, Figs. S4, S6). For α‐diversity, cation exchange capacity explained significant variation of fungal α‐diversity in organic horizons, while mean annual precipitation and soil pH explained minor amounts of variation in α‐diversity of plants and bacteria (Table 1). For β‐diversity, there were secondary influences of nutrient ratios on microbes (C:N and C:P) and K on plants (Table 2). Our data suggest that this influence of edaphic properties on microbial α‐ and β‐diversity is more significant for fungal α‐diversity and in organic horizons (Table 2, Appendix S1: Table S3). Fungi are the primary decomposers of plant‐derived lignocellulosic biomass and the upper part of the soil profile is where decomposition processes reflect the early stages of carbohydrate polymer breakdown. We know that plant litter chemistry varies with elevation along this transect as a result of the influence of MAT on plant communities (van de Weg et al. 2009, Salinas et al. 2010) and that it can affect soil microbial community composition (Orwin et al. 2010, de Vries et al. 2012, Fanin et al. 2014). Thus, elevation‐related shifts in plant litter chemistry may determine fungal α‐diversity patterns in organic horizons, and be an additional determinant of fungal β‐diversity and its coupling with plant β‐diversity.

The diversity patterns we observed may have been reinforced by biotic interactions between functional groups of plants and microbes, in addition to environmental filtering. For example, multiple lines of evidence suggest an influence of plant organic matter inputs on soil microbes, where these inputs are in turn determined by temperature effects on plant communities and productivity (van de Weg et al. 2014), for example, (1) the large difference in microbial diversity (α and β) between organic and mineral soil horizons (Appendix S1: Figs. S1, S2); (2) the stronger correlations between microbial β‐diversity and nutrients in organic horizons compared to mineral horizons (Table 2, Appendix S1: Fig. S4); (3) the overall strong correlation between plant and soil microbial diversity (Fig. 3, Appendix S1: Table S4); (4) the correlation between soil microbial β‐diversity and enzymatic activity, indices of organic nutrient degradation (Nottingham et al. 2015b) (Appendix S1: Fig. S6); (5) fungal α‐diversity in organic horizons significantly increased above the treeline, coinciding with an abrupt change in plant organic matter inputs from vegetation dominated by grassland (Fig. 2). Laboratory incubations of soils from this transect (Whitaker et al. 2014) and studies from tropical forest in French Guiana (Fanin et al. 2011, 2014) also support the link between differences in microbial community composition and organic matter inputs, and their rates of degradation. Together these findings point towards a relationship between the high soil microbial diversity in tropical forests and plant organic matter inputs to soil, through the high inter‐ and intra‐species chemical diversity in leaf litter. To further investigate the role of biotic interactions between plants and soil microbial communities in shaping these diversity patterns, further studies are required using a targeted sampling methodology by plant species or functional group (e.g., root‐associated soil microbial communities; Barberan et al. 2015, Teste et al. 2017, Leff et al. 2018).

Our results, from a 3.5‐km elevation range, contrast with previous findings from studies of elevation gradients globally that examined plant and microbial α‐diversity but did not find such strong α‐diversity correlations (Bryant et al. 2008, Fierer et al. 2011, Shen et al. 2013, 2014, Geml et al. 2014, Singh et al. 2014). The fundamental temperature–microbial‐diversity relationships we have observed were likely obscured in previous studies by the confounding influence of wider natural among‐site variation in soil pH, soil moisture, plant–host distributions (for fungi, in particular) and, in some instances, by insufficient sampling intensity or elevation range. For example, variation in bacterial diversity along a 1,850‐m elevation gradient in South Korea was related to the variation in rainfall (1713–3,743 mm) and soil pH (3.7–5.8; Singh et al. 2014), while variation in rainfall (280–3,280 mm) and soil pH also explained microbial diversity along a 950 m elevation gradient in Hawaii (Peay et al. 2017). Soil pH effects on fungal α‐diversity along mountain gradients have been found (Geml 2017), including a positive effect on the α‐diversity of AM fungi along an elevation gradient in Andean subtropical forest where soil pH varied widely (3.8–7.2; Geml et al. 2014). Fungal α‐diversity increased with a 4°C temperature increase across an elevation gradient in tropical montane cloud forest in Costa Rica, although decreased soil moisture also explained this diversity pattern (Looby et al. 2016). The majority of fungal diversity studies on mountain gradients have focused on specific phyla, reporting high variation in α‐diversity patterns (Geml et al. 2014, 2017, Merckx et al. 2015, Geml 2017) and strong associations with plant–host distributions such as with AM‐ and EM‐fungal‐associated communities (Kivlin et al. 2017); as previously outlined, these factors can partly explain differences in fungal α‐diversity patterns in organic and mineral horizons in this study. The importance of sampling intensity is demonstrated by the contrast between findings from this study of 14 sites with an earlier report from six locations along the same Andean transect where no elevation‐related gradient in soil bacterial α‐diversity was found (Fierer et al. 2011): if we reduce our data set to include only those sites represented in the earlier study, no strong elevation trends are found (Appendix S1: Fig. S7). Similarly, these factors may have accounted for the lack of clear patterns in bacterial diversity for two temperate zone elevation transect studies that sampled only six locations over 1,670 m in Northeast China (Shen et al. 2014), and five locations over 920 m in the Rocky Mountains, the latter indicating a single‐taxon increase with elevation, but no community‐wide trend (Bryant et al. 2008). Last, the detection of these elevation–diversity patterns may also depend on the length and, therefore, temperature range of the transect. For example, the absence of bacterial diversity patterns along a 900 m gradient in tropical montane forest in Hawaii may have been because the 5°C temperature difference did not substantially affect plant community composition (Selmants et al. 2016). In contrast, the temperature‐driven diversity patterns in bacteria and fungi demonstrated for this large Peruvian gradient (20°C difference in MAT) likely resulted, in part, from indirect temperature effects on plant communities, thus contributing to the correlated diversity patterns among these three biotic groups.

This elevation gradient study in the Peruvian Andes demonstrates how temperature fundamentally shapes plant, bacterial and fungal diversity in tropical forests, whether directly for each group, or indirectly for microbial groups through temperature effects on plant communities and productivity. Consistent trends in both α‐ and β‐diversity were observed across the principal organismal groups of plants, bacteria and fungi, suggesting that stronger interactions occur among these groups than has been recognized previously. The role of temperature in driving these coordinated patterns was revealed by the occurrence in our study transect of a narrow natural range in soil pH and moisture, and by intensive sampling across space, and in separate soil horizons. We suggest that this relationship will be obscured across unconstrained environmental gradients often associated elsewhere with differences in elevation and latitude (Fierer and Jackson 2006, Bryant et al. 2008, Tedersoo et al. 2014), and its detection is further hindered by shallower diversity gradients for soil microbes compared to plants (Figs. 2, 3; Tedersoo et al. 2014, Zhou et al. 2016). Our findings imply that, where other influences such as soil pH and moisture remain relatively constrained, anticipated future temperature change will have significant coordinated and fundamental impacts on the identity and functioning of tropical biota both above and below‐ground.

## Data Availability

Data are available on Figshare: https://doi.org/10.6084/m9.figshare.6843701.v1.
