# Supplementary material for: Microbes follow Humboldt: temperature drives plant and soil microbial diversity patterns from the Amazon to the Andes
Source: Ecology. 2018 Oct 26;99(11):2455–66. doi: 10.1002/ecy.2482 (PMC6850070; doi:10.1002/ecy.2482)
Supplement: Supplementary file 1 [file ECY-99-2455-s001.pdf]

Supporting Information. Microbes follow Humboldt: temperature drives plant and soil microbial diversity patterns from the Amazon to the Andes. Andrew T. Nottingham, Noah Fierer, Benjamin L. Turner, Jeanette Whitaker, Nick J. Ostle, Niall P. McNamara, Richard D. Bardgett, Jonathan W. Leff, Norma Salinas, Miles Silman, Loeske Kruuk, and Patrick Meir. *Ecology*. 2018.

Appendix S1

Supplementary methods: DNA sequencing

For each soil sample, DNA was extracted using the MoBio PowerSoil DNA isolation kit (MoBio Laboratories, Carlsbad, CA) following manufacturer instructions. Primers were modified to incorporate 12 bp error-correcting barcodes, and 16S rRNA amplicons and ITS amplicons were pooled separately prior to sequencing with two separate runs on an Illumina MiSeq instrument (San Diego, CA, USA) at the University of Colorado BioFrontiers Institute Next Generation Sequencing Facility. Raw sequence data were processed using the QIIME v1.7 pipeline, where sequences were de-multiplexed using their unique barcode specific to individual samples and assigned to phylotypes (operational taxonomic units, OTUs, at 97% similarity) using the 'open reference' clustering approach recommended in the pipeline (Caporaso et al. 2012). Samples were rarefied to 1,850 and 100 sequences per sample for bacteria and fungi, respectively. Rarefaction depths were chosen to balance the number of samples that could be included while maximizing the available number of sequences per sample. Although the lower rarefaction depth for fungi was a shortcoming of the study, importantly it was sufficient to characterise diversity and community composition characteristics; the diversity patterns we observed were highly correlated to those found when using higher rarefaction depth (300; $r = 0.98$) (as also found by Peay et al. (2017) for an elevation transect in Hawai'i). Taxonomy was determined for each phylotype using the RDP classifier (Wang et al. 2007) trained on the Greengenes (McDonald et al. 2012) and UNITE (Abarenkov et al. 2010) databases for bacterial and fungal sequences. Relatively abundant phylotypes were checked using BLAST and comparison against sequences contained within GenBank.

Supplementary methods: determination of soil properties

Total C and N were determined for dried, ground soil samples using a TruSpec CN Elemental Determinator (LECO, USA). Total P was determined by ignition (550°C, 1 h)

followed by extraction in 1 M H_2SO_4, with phosphate detection in neutralised extracts at 880 nm by automated molybdate colorimetry using a Lachat Quikchem 8500 (Hach Ltd, Loveland, CO, USA). Mineral N and P availability were determined using ion exchange resins (Nottingham et al. 2015). Other organic and inorganic phosphorus fractions were determined using a modification of Hedley sequential extraction (in 1M NaOH, 1M HCl) (Hedley et al. 1982) and exchangeable cations were extracted in 0.1 M BaCl (Hendershot and Duquette 1986). Soil pH was determined in H_2O (soil solution, 1:2.5 w:v). Gravimetric moisture content, bulk density (dried for 24 h at 105 °C) and water holding capacity (the amount of water remaining in the soil after being saturated and left to drain for 12 h) were calculated for composite soil samples for each site.

Enzyme activities for seven enzymes involved in carbon and nutrient cycling were determined for the 14 sites, using microplate fluorimetric assays with 100 µM methylumbelliferone (MU)-linked substrates to measure activity of β-glucosidase (degradation of β -bonds in glucose), cellobiohydrolase (degradation of cellulose), *N*-acetyl β-glucosaminidase (degradation of *N*-glycosidic bonds), phosphomonoesterase (degradation of monoester-linked simple organic phosphates) and β-xylanase (degradation of hemicellulose). Phenol oxidase (degradation of phenolic compounds) was measured using 5 mM L-dihydroxyphenyalanine (L-DOPA) as substrate. Further information on protocols for enzyme analyses is reported elsewhere (Nottingham et al. 2015).

Supplementary methods: further detail on statistical analyses

Effects of elevation on α- and β-diversity: We evaluated the change in α-diversity in each biotic group with elevation using linear and mixed effects models, including a quadratic term to test for non-linear effects of elevation. Linear models were sufficient to represent the change in plant α-diversity with elevation, but mixed effects models were required to account for the multiple measures per site for fungal and bacterial diversity. The fixed effects were elevation and its quadratic term in all models. Mixed models were run using the R-package lmerTest, with site (a 14-level factor) fitted as a random effect in every model, and using maximum likelihood to assess the significance of the fixed effects. Elevation was mean-centred for these analyses, so that the test of the significance of the linear term for elevation was not affected by the presence of the quadratic term; also, for ease of interpretation of parameter estimates, we used elevation (m a.s.l.)/1000. We report R^2 values for all models: for the mixed models, we report both the conditional (considering just the fixed effects) and

the marginal (fixed and random effects) R^2 values, calculated using the R-package piecewiseSEM (Nakagawa and Schielzeth 2013). We also fitted a linear mixed model with lmerTest to data from all five groups combined, to test for differences in their average α-diversity between groups and in the change with elevation; these differences were assessed by their F statistics and Satterthwaite approximation for degrees of freedom.

Elevational differences in β-diversity were examined using Permutational MANOVA (PERMANOVA) with the dissimilarity matrix of relative abundances ('community composition') as the fixed factor and elevation as the random factor; the same approach was used to determine bacterial and fungal β-diversity differences between soil horizons. Further trends were identified using principal coordinates analyses and, for a specific Phylum or Class, using Spearman correlations of abundance against elevation.

Effects of climate and edaphic variables on α-diversity: We tested for effects of seven specific variables on α-diversity: MAT, distance (relative spatial separation of plots in km), MAP, pH, total C, ECEC and resin P. We selected these seven variables because we predicted that they were most likely to have effects on plant and soil microbial diversity, based on previous studies indicating influences of geography, climate and soil pH and fertility (Fierer and Jackson 2006, Tedersoo et al. 2014). As above, this involved using a linear model for plant diversity, and linear mixed models for each of the four measures of soil microbial diversity. We started with full models with all seven variables as fixed effects, and reduced these to final models containing only significant variables. To investigate covariance among the variables in the final models we used Variance Inflation Factors (VIFs), where VIF > 5 indicated covariance between terms. We report the full models in the Supplementary Information, and the final models in the main text.

To test for the effects of climate and edaphic variables on β-diversity, we used the BIO-ENV multivariate correlation method (Clarke and Ainsworth 1993), which creates a model by determining high-rank correlations between species dissimilarity matrices and resemblance matrices generated from environmental variables. The method used step-wise selection to determine the suite of variables that best explain the biotic structure, starting with a full set of climate and edaphic variables (31 in total). To address potential covariance among the environmental variables we followed Clarke and Ainsworth (1993), by identifying highly correlated pairs of variables (at $\rho > 0.95$) and removing one from each pair, prior to model-fitting. As an *ad-hoc* exploration of the significance of individual parameters in the

final model, we used Mantel tests between species and predictor variable matrices. For these, and for all subsequent mantel tests we report Spearman correlation coefficients between the two matrices (ρ).

Correlations between α- and β-diversity of different groups: For α-diversity we estimated the pairwise Pearson correlations between the five groups (plants, and fungi or bacteria in mineral or organic horizons), using the averages of the three measures for each site for the microbial data. For β-diversity we used Mantel tests between species dissimilarity matrices.

Soil β-diversity and function: To investigate whether there was a relationship between soil microbial community composition and function we used Mantel tests between Bray-Curtis dissimilarity matrices for microbial community composition (for either bacteria or fungi in either organic or mineral soils) and Bray-Curtis dissimilarity matrices for enzymatic activity (across the seven enzymes).

Abarenkov, K., R. H. Nilsson, K. H. Larsson, I. J. Alexander, U. Eberhardt, S. Erland, K. Hoiland, R. Kjoller, E. Larsson, T. Pennanen, R. Sen, A. F. S. Taylor, L. Tedersoo, B. M. Ursing, T. Vralstad, K. Liimatainen, U. Peintner, and U. Koljalg. 2010. The UNITE database for molecular identification of fungi - recent updates and future perspectives. New Phytologist **186**:281-285.

Caporaso, J. G., C. L. Lauber, W. A. Walters, D. Berg-Lyons, J. Huntley, N. Fierer, S. M. Owens, J. Betley, L. Fraser, M. Bauer, N. Gormley, J. A. Gilbert, G. Smith, and R. Knight. 2012. Ultra-high-throughput microbial community analysis on the Illumina HiSeq and MiSeq platforms. Isme Journal **6**:1621-1624.

Clarke, K. R., and M. Ainsworth. 1993. A Method of Linking Multivariate Community Structure to Environmental Variables. Marine Ecology Progress Series **92**:205-219.

Fierer, N., and R. B. Jackson. 2006. The diversity and biogeography of soil bacterial communities. Proceedings of the National Academy of Sciences of the United States of America **103**:626-631.

Hedley, M. J., J. W. B. Stewart, and B. S. Chauhan. 1982. Changes in Inorganic and Organic Soil-Phosphorus Fractions Induced by Cultivation Practices and by Laboratory Incubations. Soil Science Society of America Journal **46**:970-976.

Hendershot, W. H., and M. Duquette. 1986. A Simple Barium-Chloride Method for Determining Cation-Exchange Capacity and Exchangeable Cations. Soil Science Society of America Journal **50**:605-608.

McDonald, D., M. N. Price, J. Goodrich, E. P. Nawrocki, T. Z. DeSantis, A. Probst, G. L. Andersen, R. Knight, and P. Hugenholtz. 2012. An improved Greengenes taxonomy with explicit ranks for ecological and evolutionary analyses of bacteria and archaea. Isme Journal **6**:610-618.

Nakagawa, S., and H. Schielzeth. 2013. A general and simple method for obtaining R2 from generalized linear mixed-effects models. Methods in Ecology and Evolution **4**:133-142.

Nottingham, A. T., B. L. Turner, J. Whitaker, N. Ostle, N. P. McNamara, R. D. Bardgett, N. Salinas, and P. Meir. 2015. Soil microbial nutrient constraints along a tropical forest elevation gradient: a belowground test of a biogeochemical paradigm. Biogeosciences **12**:6489-6523.

Peay, K. G., C. von Sperber, E. Cardarelli, H. Toju, C. A. Francis, O. A. Chadwick, and P. M. Vitousek. 2017. Convergence and contrast in the community structure of Bacteria, Fungi and Archaea along a tropical elevation-climate gradient. Fems Microbiology Ecology **93**.

Tedersoo, L., M. Bahram, S. Polme, U. Koljalg, N. S. Yorou, R. Wijesundera, L. V. Ruiz, A. M. Vasco-Palacios, P. Q. Thu, A. Suija, M. E. Smith, C. Sharp, E. Saluveer, A. Saitta, M. Rosas, T. Riit, D. Ratkowsky, K. Pritsch, K. Poldmaa, M. Piepenbring, C. Phosri, M. Peterson, K. Parts, K. Partel, E. Otsing, E. Nouhra, A. L. Njouonkou, R. H. Nilsson, L. N. Morgado, J. Mayor, T. W. May, L. Majuakim, D. J. Lodge, S. S. Lee, K. H. Larsson, P. Kohout, K. Hosaka, I. Hiiesalu, T. W. Henkel, H. Harend, L. D. Guo, A. Greslebin, G. Grelet, J. Geml, G. Gates, W. Dunstan, C. Dunk, R. Drenkhan, J. Dearnaley, A. De Kesel, T. Dang, X. Chen, F. Buegger, F. Q. Brearley, G. Bonito, S. Anslan, S. Abell, and K. Abarenkov. 2014. Global diversity and geography of soil fungi. Science **346**:1078.

Wang, Q., G. M. Garrity, J. M. Tiedje, and J. R. Cole. 2007. Naive Bayesian classifier for rapid assignment of rRNA sequences into the new bacterial taxonomy. Applied and Environmental Microbiology **73**:5261-5267.

Figure S1A. The relative abundances of fungal taxa in organic and mineral horizons in Andean tropical forest soils, where red indicates high and blue indicates low relative abundance. The sites are in order of dissimilarity of communities among sites, whereby sites closer together are more similar in community composition.

Organic

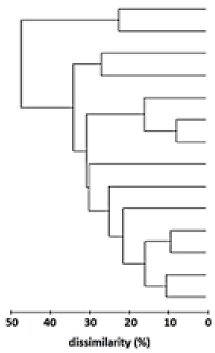

kingdom	Fungi																
phyla	Ascomycota						Basidiomycota				Chytridiomycota		Glomeromycota	Zygomycota		*Other*
class	*Archaeorhizomycetes*	*Dothideomycetes*	*Eurotiomycetes*	*Leotiomycetes*	*Sordariomycetes*	*Other*	*Agaricomycetes*	*Microbotryomycetes*	*Tremellomycetes*	*Other*	*Chytridiomycetes*	*Other*		*Incertae sedis*	*Other*	
**194**	0.00	0.03	0.09	0.00	0.42	0.17	0.08	0.01	0.00	0.02	0.00	0.01	0.00	0.08	0.00	0.09
**210**	0.06	0.03	0.05	0.01	0.29	0.20	0.09	0.00	0.01	0.02	0.00	0.00	0.01	0.04	0.13	0.07
**1000**	0.14	0.00	0.01	0.01	0.06	0.32	0.03	0.00	0.00	0.01	0.00	0.00	0.00	0.11	0.22	0.10
**1500**	0.03	0.00	0.01	0.01	0.08	0.49	0.04	0.00	0.10	0.01	0.00	0.00	0.00	0.04	0.14	0.06
**2520**	0.32	0.00	0.00	0.02	0.01	0.45	0.04	0.00	0.00	0.02	0.00	0.00	0.00	0.05	0.01	0.06
**1850**	0.20	0.00	0.00	0.06	0.04	0.52	0.04	0.00	0.00	0.00	0.00	0.00	0.00	0.01	0.03	0.08
**2020**	0.23	0.00	0.02	0.04	0.02	0.52	0.02	0.00	0.00	0.00	0.00	0.00	0.00	0.01	0.01	0.13
**2720**	0.45	0.00	0.00	0.04	0.02	0.28	0.06	0.00	0.01	0.02	0.00	0.01	0.00	0.01	0.00	0.09
**3400**	0.15	0.00	0.01	0.03	0.10	0.22	0.18	0.01	0.12	0.02	0.00	0.01	0.00	0.08	0.00	0.08
**3025**	0.32	0.00	0.00	0.09	0.15	0.24	0.07	0.01	0.03	0.02	0.00	0.01	0.00	0.03	0.00	0.03
**3020**	0.20	0.01	0.00	0.05	0.09	0.27	0.06	0.00	0.03	0.01	0.00	0.02	0.00	0.21	0.00	0.05
**3200**	0.24	0.00	0.02	0.02	0.10	0.29	0.04	0.00	0.05	0.01	0.00	0.01	0.00	0.16	0.00	0.07
**1750**	0.24	0.00	0.01	0.01	0.12	0.35	0.02	0.00	0.01	0.04	0.01	0.00	0.00	0.07	0.06	0.05
**3644**	0.24	0.01	0.00	0.05	0.07	0.33	0.02	0.02	0.01	0.01	0.00	0.00	0.00	0.10	0.04	0.09

Mineral

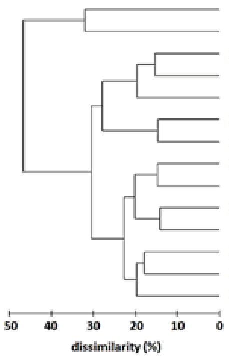

kingdom	Fungi															
phyla	Ascomycota						Basidiomycota				Chytridiomycota		Glomeromycota	Zygomycota		*Other*
class	*Archaeorhizomycetes*	*Dothideomycetes*	*Eurotiomycetes*	*Leotiomycetes*	*Sordariomycetes*	*Other*	*Agaricomycetes*	*Microbotryomycetes*	*Tremellomycetes*	*Other*	*Chytridiomycetes*	*Other*		*Incertae sedis*	*Other*	
**210**	0.07	0.02	0.04	0.01	0.15	0.24	0.13	0.00	0.02	0.06	0.01	0.00	0.01	0.01	0.15	0.09
**194**	0.04	0.02	0.06	0.00	0.37	0.23	0.08	0.00	0.02	0.02	0.00	0.00	0.00	0.09	0.01	0.06
**3025**	0.25	0.00	0.02	0.02	0.00	0.64	0.03	0.01	0.00	0.00	0.00	0.00	0.00	0.01	0.00	0.02
**1850**	0.21	0.00	0.02	0.02	0.03	0.53	0.04	0.00	0.00	0.00	0.00	0.00	0.00	0.03	0.01	0.09
**1750**	0.10	0.00	0.00	0.03	0.03	0.61	0.04	0.00	0.00	0.01	0.00	0.00	0.00	0.01	0.10	0.05
**3644**	0.36	0.00	0.00	0.04	0.04	0.36	0.00	0.01	0.00	0.04	0.00	0.00	0.00	0.07	0.01	0.07
**2720**	0.36	0.00	0.01	0.01	0.02	0.47	0.01	0.00	0.01	0.00	0.00	0.00	0.00	0.04	0.02	0.06
**3200**	0.17	0.00	0.00	0.05	0.07	0.30	0.12	0.01	0.00	0.00	0.01	0.00	0.00	0.12	0.08	0.07
**1000**	0.10	0.04	0.00	0.02	0.07	0.32	0.09	0.00	0.01	0.01	0.00	0.00	0.00	0.13	0.15	0.07
**3020**	0.17	0.00	0.00	0.02	0.06	0.42	0.08	0.00	0.01	0.01	0.00	0.00	0.00	0.08	0.05	0.09
**1500**	0.15	0.02	0.01	0.01	0.10	0.42	0.02	0.00	0.01	0.02	0.01	0.00	0.00	0.09	0.09	0.06
**2520**	0.21	0.00	0.00	0.02	0.05	0.33	0.01	0.00	0.00	0.01	0.01	0.00	0.00	0.25	0.03	0.08
**2020**	0.24	0.01	0.01	0.01	0.03	0.44	0.00	0.00	0.01	0.01	0.00	0.00	0.00	0.14	0.02	0.08
**3400**	0.22	0.00	0.00	0.02	0.04	0.34	0.12	0.02	0.02	0.01	0.00	0.01	0.00	0.10	0.00	0.10

Figure S1B. The relative abundances of bacterial taxa in organic and mineral horizons in Andean tropical forest soils, where red indicates high and blue indicates low relative abundance. The sites are in order of dissimilarity of communities among sites, whereby sites closer together are more similar in community composition.

Organic

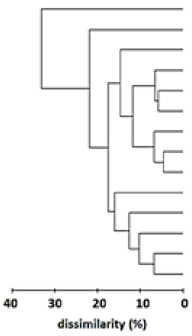

kingdom	Archaea		Bacteria														
phyla	Crenarchaeota	Euryarchaeota	Acidobacteria	Actinobacteria	Bacteroidetes	Chloroflexi	Firmicutes	Gemmatimonadetes	Nitrospirae	Planctomycetes	Proteobacteria					Verrucomicrobia	Other/Unclassified
class											*Alphaproteobacteria*	*Betaproteobacteria*	*Deltaproteobacteria*	*Gammaproteobacteria*	*Other*		
**194**	0.00	0.00	0.17	0.08	0.05	0.02	0.03	0.03	0.03	0.03	0.23	0.11	0.07	0.09	0.00	0.04	0.03
**3400**	0.00	0.01	0.34	0.03	0.01	0.00	0.00	0.00	0.00	0.02	0.14	0.28	0.02	0.11	0.00	0.01	0.02
**1500**	0.01	0.01	0.32	0.06	0.02	0.01	0.01	0.01	0.00	0.03	0.18	0.08	0.06	0.13	0.01	0.03	0.04
**1750**	0.01	0.02	0.40	0.05	0.02	0.00	0.01	0.01	0.00	0.03	0.15	0.09	0.03	0.12	0.00	0.03	0.03
**2520**	0.01	0.01	0.43	0.03	0.03	0.00	0.00	0.00	0.00	0.04	0.13	0.09	0.03	0.12	0.01	0.04	0.04
**2720**	0.00	0.01	0.40	0.04	0.03	0.00	0.00	0.01	0.00	0.05	0.12	0.07	0.03	0.14	0.01	0.04	0.04
**3025**	0.00	0.01	0.37	0.07	0.01	0.00	0.00	0.01	0.00	0.03	0.15	0.15	0.02	0.12	0.00	0.02	0.03
**3020**	0.01	0.03	0.38	0.03	0.02	0.00	0.01	0.01	0.00	0.02	0.14	0.16	0.03	0.12	0.00	0.03	0.03
**3200**	0.01	0.00	0.38	0.02	0.02	0.00	0.00	0.01	0.00	0.03	0.16	0.17	0.02	0.12	0.00	0.02	0.03
**210**	0.01	0.00	0.40	0.09	0.02	0.01	0.00	0.01	0.00	0.02	0.21	0.03	0.03	0.09	0.00	0.03	0.04
**3644**	0.00	0.00	0.45	0.04	0.02	0.01	0.00	0.00	0.00	0.04	0.18	0.06	0.05	0.05	0.00	0.06	0.03
**1000**	0.02	0.01	0.52	0.03	0.01	0.01	0.00	0.01	0.00	0.01	0.15	0.03	0.04	0.08	0.01	0.03	0.03
**1850**	0.01	0.01	0.49	0.04	0.02	0.00	0.01	0.01	0.00	0.05	0.13	0.07	0.03	0.06	0.01	0.03	0.03
**2020**	0.00	0.01	0.49	0.04	0.02	0.00	0.00	0.00	0.00	0.05	0.14	0.04	0.05	0.08	0.01	0.04	0.03

Mineral

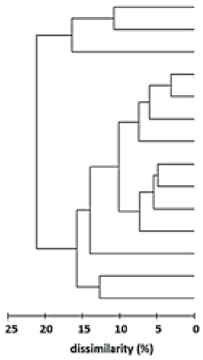

kingdom	Archaea		Bacteria														
phyla	Crenarchaeota	Euryarchaeota	Acidobacteria	Actinobacteria	Bacteroidetes	Chloroflexi	Firmicutes	Gemmatimonadetes	Nitrospirae	Planctomycetes	Proteobacteria					Verrucomicrobia	Other/Unclassified
class											*Alphaproteobacteria*	*Betaproteobacteria*	*Deltaproteobacteria*	*Gammaproteobacteria*	*Other*		
**1850**	0.01	0.01	0.49	0.04	0.02	0.00	0.01	0.01	0.00	0.05	0.13	0.07	0.03	0.06	0.01	0.03	0.03
**210**	0.01	0.00	0.40	0.09	0.02	0.01	0.00	0.01	0.00	0.02	0.21	0.03	0.03	0.09	0.00	0.03	0.04
**194**	0.00	0.00	0.17	0.08	0.05	0.02	0.03	0.03	0.03	0.03	0.23	0.11	0.07	0.09	0.00	0.04	0.03
**2520**	0.01	0.01	0.43	0.03	0.03	0.00	0.00	0.00	0.00	0.04	0.13	0.09	0.03	0.12	0.01	0.04	0.04
**1750**	0.01	0.02	0.40	0.05	0.02	0.00	0.01	0.01	0.00	0.03	0.15	0.09	0.03	0.12	0.00	0.03	0.03
**1000**	0.02	0.01	0.52	0.03	0.01	0.01	0.00	0.01	0.00	0.01	0.15	0.03	0.04	0.08	0.01	0.03	0.03
**1500**	0.01	0.01	0.32	0.06	0.02	0.01	0.01	0.01	0.00	0.03	0.18	0.08	0.06	0.13	0.01	0.03	0.04
**3200**	0.01	0.00	0.38	0.02	0.02	0.00	0.00	0.01	0.00	0.03	0.16	0.17	0.02	0.12	0.00	0.02	0.03
**3020**	0.01	0.03	0.38	0.03	0.02	0.00	0.01	0.01	0.00	0.02	0.14	0.16	0.03	0.12	0.00	0.03	0.03
**2720**	0.00	0.01	0.40	0.04	0.03	0.00	0.00	0.01	0.00	0.05	0.12	0.07	0.03	0.14	0.01	0.04	0.04
**2020**	0.00	0.01	0.49	0.04	0.02	0.00	0.00	0.00	0.00	0.05	0.14	0.04	0.05	0.08	0.01	0.04	0.03
**3400**	0.00	0.01	0.34	0.03	0.01	0.00	0.00	0.00	0.00	0.02	0.14	0.28	0.02	0.11	0.00	0.01	0.02
**3644**	0.00	0.00	0.45	0.04	0.02	0.01	0.00	0.00	0.00	0.04	0.18	0.06	0.05	0.05	0.00	0.06	0.03
**3025**	0.00	0.01	0.37	0.07	0.01	0.00	0.00	0.01	0.00	0.03	0.15	0.15	0.02	0.12	0.00	0.02	0.03

Figure S2. Principal Co-ordinates Analysis of A) bacterial and B) Fungal β-diversity in organic and mineral horizons. β-diversity differed between organic and mineral soils for bacteria ($p < 0.001$, F = 8.0, DF = 82) and fungi ($p < 0.001$, F = 3.2, df = 83; by PERMANOVA).

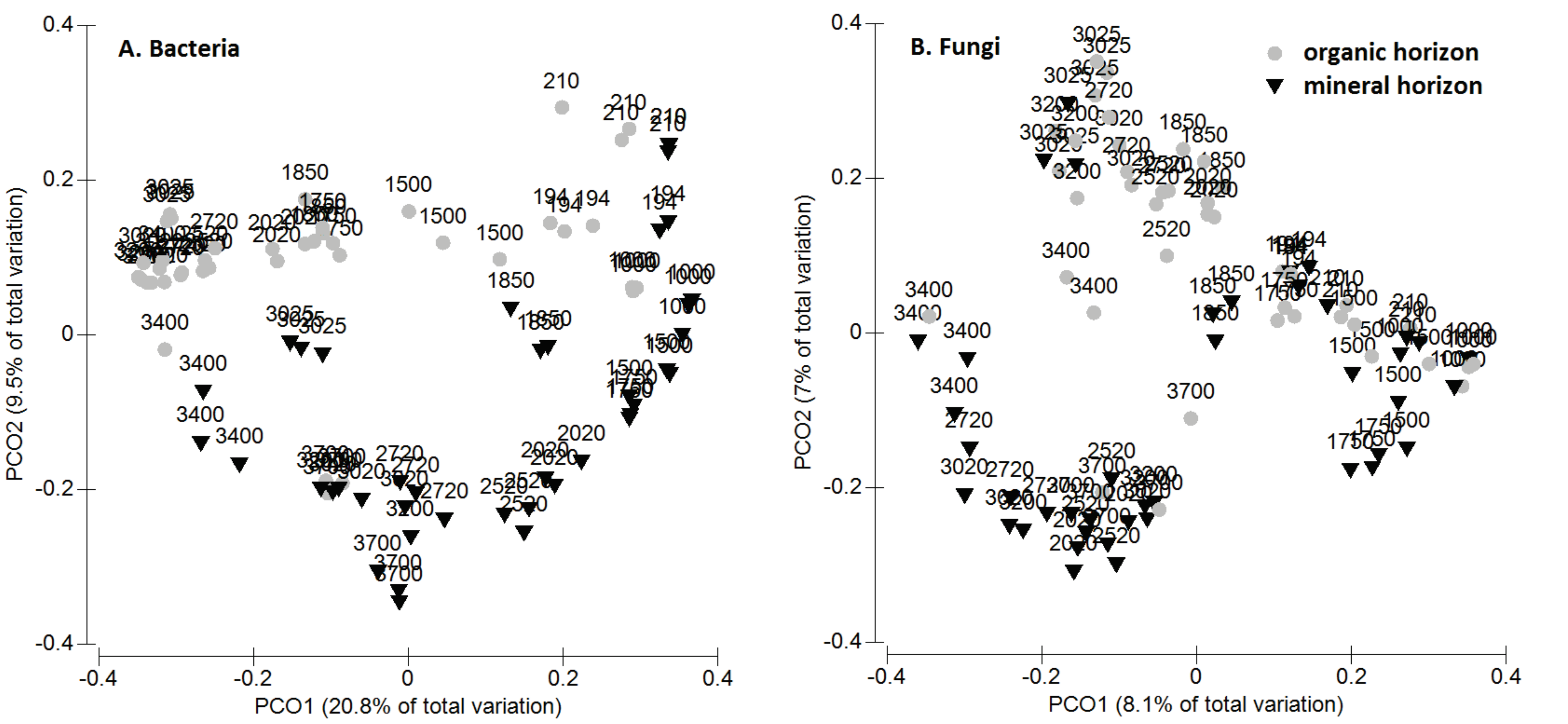

Figure S3. The (A) positive and (B) negative trends in the relative abundances of specific bacterial and fungal taxa in soils along an elevation gradient in Andean tropical forest. Bacteria taxa are Acidobacteria, Actinobacteria, *Beta-Proteobacteria* and *Delta-Proteobacteria*; and fungal taxa are Actinomycetes, *Sordariomycetes* and *Archaeorhizomycetes* (by phyla or, where italicised, by class). All data are for organic horizons except for Acidobacteria, which is for mineral horizon. The full data for all taxa in organic and mineral horizons (which follow similar trends) are in Fig. S1 and Table S5 (Fig. S1 shows dissimilarity of communities among sites using heat-maps and Table S5 shows correlations between relative abundance of taxa and elevation).

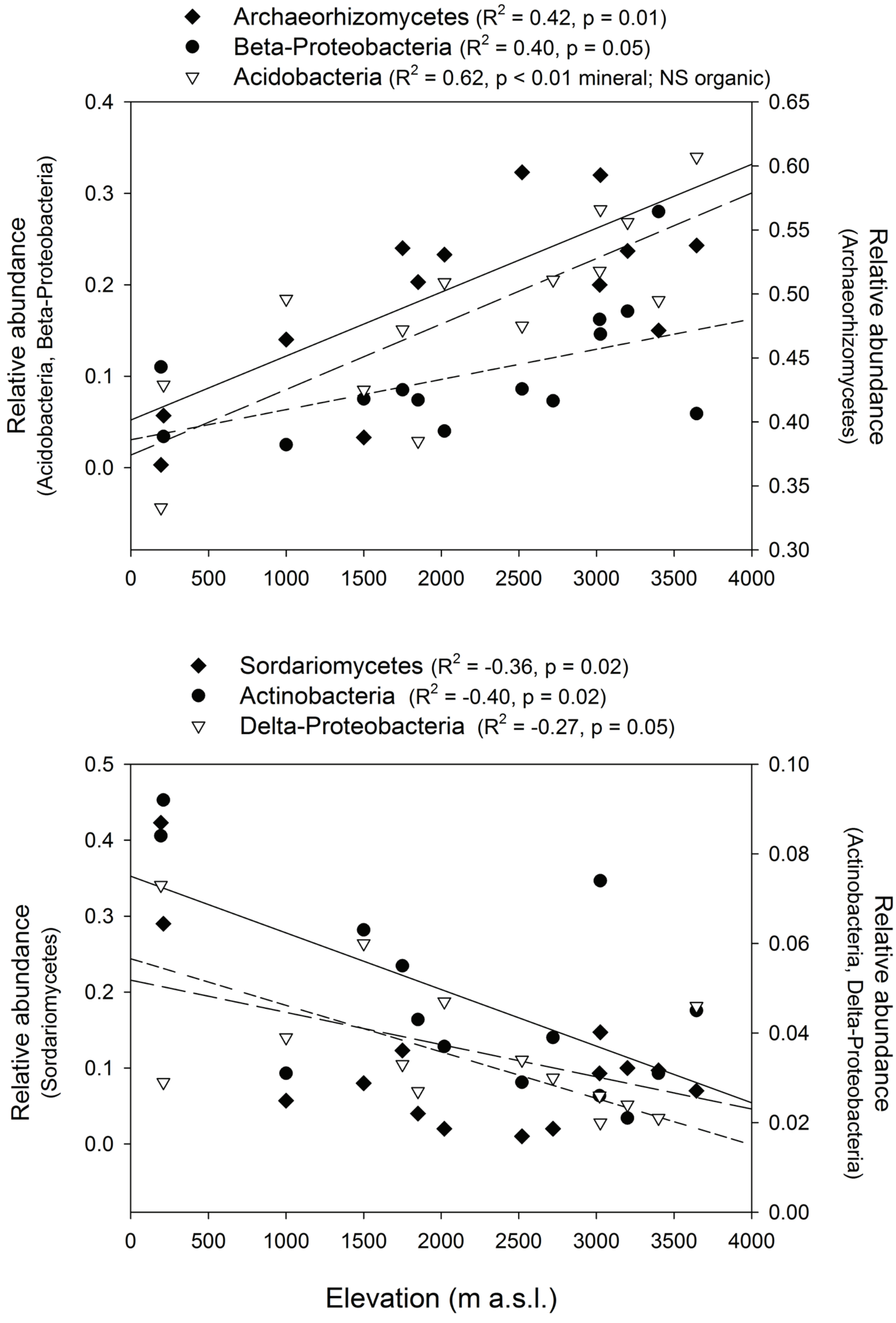

Figure S4. The relationships between the β-diversity of A) bacteria, and B) fungi, and climatic and edaphic factors. Data are shown for mineral (light grey) organic (dark grey) horizons. Values are results from Mantel tests for community composition and each property (Spearman correlation coefficients between Bray-Curtis dissimilarity matrices).

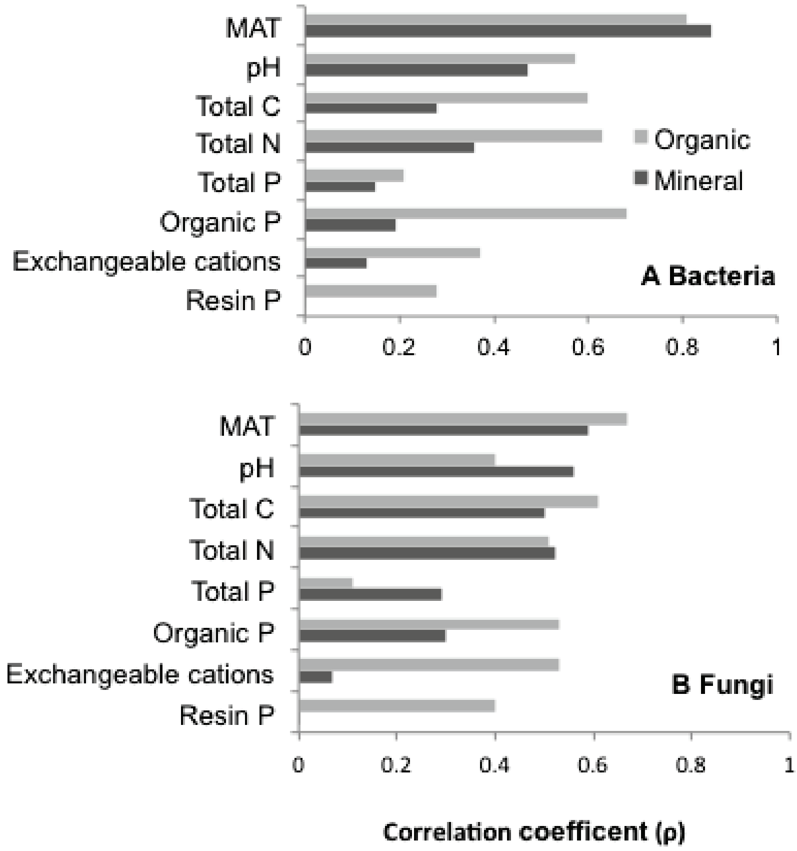

Figure S5. Enzyme activities (V_{max}), for carbon (β-glucosidase, cellobiohydrolase, β-xylanase, phenol oxidase), nitrogen (*N*-acetyl β-glucosaminidase), phosphorus (phosphomonoesterase) and sulfur (sulfatase) - degrading enzymes. Enzymes were determined at the mean annual temperature (MAT) for each site (Table 1). Enzyme activity at MAT was determined using linear regression of temperature and maximum potential enzyme activities determined at 2, 10 , 22 and 30 ºC (Table S1). Refer to Nottingham et al. (2015) for further analytical detail. The vertical dashed line represents the approximate location of the tree-line. Values are means with 1 SE (n = 5 replicates, which represents the spatial variation within a 1 ha plot).

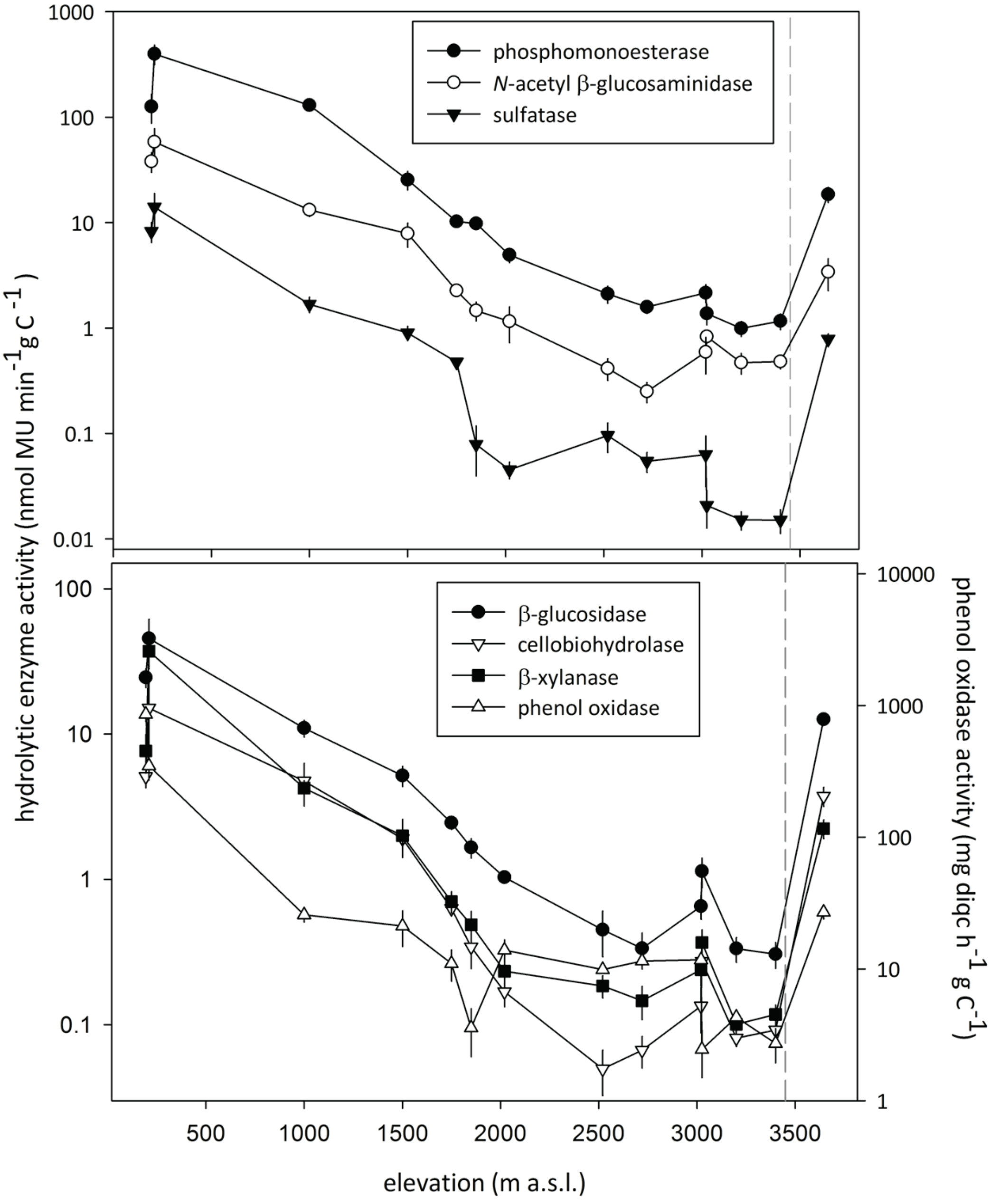

Figure S6. Relating microbial β-diversity to soil process rates. The relationships between Bray–Curtis dissimilarity matrices of bacterial and fungal β-diversity (determined by Mantel tests: pair-wise differences in dissimilarity matrices of OTUs and extracellular enzyme activities in organic horizons.

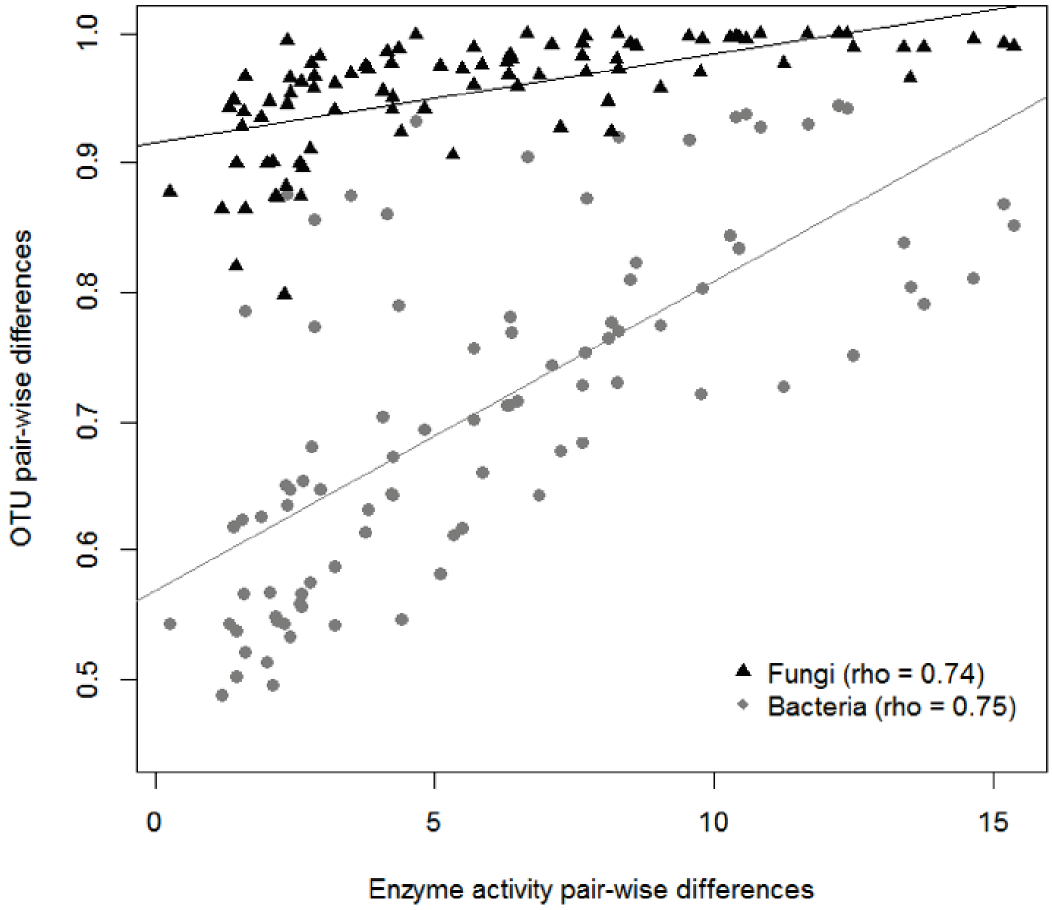

Figure S7. The α-diversity (Shannon diversity index) of bacteria and fungi in organic and mineral soil horizons across the elevation gradient, using only the sites represented in Fierer et al. (2011). In contrast to the strong relationships we found across 14 sites in this study (Fig. 2), when we reduce the number of sites included in the analyses the gradients in diversity are weak or not significant (ns).

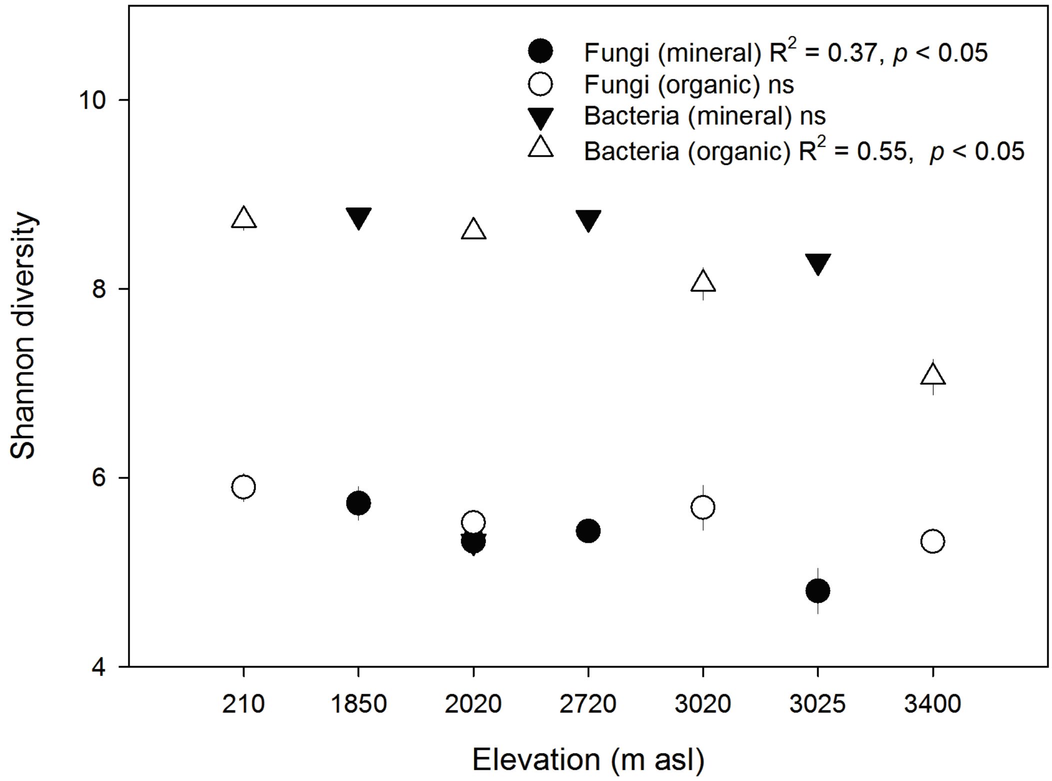

Table S1. Summary of site characteristics and soil chemical and physical properties (in mineral horizons) along the elevation gradient.

Vegetation	Site code	Elevation (m asl)	Latitude	Longitude	Distance (km relative to TC)	Mean annual air temp (MAT) (^{o}C)	Annual precipitation (MAP) (mm yr^{-1})	Soil organic horizon (cm)	Soil pH	Total carbon (%)	Total nitrogen (%)	Total phosphorus (mg g^{-1})	Aspect (deg)	Slope (deg)	Parent material
Lowland rainforest	TAM-06	194	-12.839	-69.296	253.9	26.4	1900	0.7	4.6	3.0	0.40	0.52	169.4	4	Holocene alluvial terrace
	TAM-05	210	-12.830	- 69.271	256.7	26.4	1900	2.5	3.7	2.1	0.27	0.25	186.2	6.9	Pleistocene alluvial terrace
Pre-montane rainforest rainforest	TON-02	1000	-12.866	-71.401	36.9	21.3	3100	3.0	4.1	4.9	0.35	0.75	NA	NA	NA
Lower montane rainforest	SPD-2	1500	-13.049	- 71.537	12.0	17.4	5302	16.0	3.9	4.8	0.27	1.44	143.5	39	Plutonic intrusion (granite)
"	SPD-1	1750	-13.047	- 71.543	11.7	15.8	5302	9.6	3.9	7.0	0.58	1.94	141.9	40.1	Plutonic intrusion (granite)
"	TRU-08	1850	-13.071	-71.555	8.9	16.0	2472	15.6	4.1	10.1	0.60	0.40	137.0	41.8	Plutonic intrusion (granite)
"	TRU-07	2020	-13.074	-71.559	8.4	14.9	1827	16.8	3.9	13.2	0.83	0.62	NA	NA	Paleozoic shales-slates /Granite
Upper montane rainforest	TRU-05	2520	-13.094	-71.574	5.8	12.1	2318	13.6	3.8	15.6	1.07	0.31	NA	NA	Paleozoic shales-slates
	TRU-04	2720	-13.107	- 71.589	3.6	11.1	2318	21.4	4.0	3.7	0.33	0.96	189.8	28.6	Paleozoic shales-slates
"	TRU-03	3020	-13.109	-71.600	2.5	9.5	1776	17.2	4.3	10.5	0.78	1.06	129.3	37.6	Paleozoic shales-slates
"	WAY-01	3045	-13.190	-71.587	8.1	11.1	1506	22.8	4.1	14.6	0.91	1.32	NA	NA	Paleozoic shales-slates
"	TRU-02	3200	-13.111	-71.604	2.0	8.9	2555	11.8	4.0	10.9	0.77	1.05	NA	NA	Paleozoic shales-slates
"	TRU-01	3400	-13.114	-71.607	1.6	7.7	2555	14.0	4.0	11.5	0.81	1.52	144.3	34.3	Paleozoic shales-slates
Grassland	TC	3644	-13.123	-71.618	0	6.5	760	4	4.5	9.2	0.82	0.59	NA	NA	Paleozoic shales-slates

Values are means with 1 SE (n = 5). NA = data not available

Table S2. Effect of elevation on α-diversity. Linear model for plants (n = 13) and linear mixed models for fungi/bacteria (with Site as a random effect; n = 42). Elevation was fitted as m.s.l./1000, and was mean-centred. Marginal R^2 gives the proportion of variance accounted for by the fixed effects; conditional R^2 that accounted for by both fixed and random effects (Nakagawa and Schielzeth 2013). We checked for spatial autocorrelation of the residuals for each model using Moran's I index (not significant in all cases)

(a) Plants				
	Parameter	Std. Error	t-value	p-value
Intercept	3.652	0.111	32.824	0
Elevation	-0.753	0.094	-8.033	0
Elevation^2	-0.131	0.079	-1.666	0.127
R^2	0.890			
(b) Fungal organic				
	Estimate	Std. Error	t-value	p-value
Intercept	5.156	0.108	47.777	0
Elevation	-0.03	0.076	-0.396	0.698
Elevation^2	0.172	0.067	2.557	0.023
Random effects variance				
Site	0.05361			
Residual	0.06604			
Marginal R^2	0.308	Conditional R^2	0.6178028	
(c) Fungal mineral				
	Estimate	Std. Error	t value	p-value
Intercept	5.303	0.09	59.071	0
Elevation	-0.182	0.063	-2.878	0.012
Elevation^2	0.026	0.056	0.466	0.648
Random effects variance				
Site	0.03590			
Residual	0.04925			
Marginal R^2	0.355	Conditional R^2	0.627	
(d) Bacterial organic				
	Estimate	Std. Error	t value	p-value
Intercept	8.575	0.136	63.028	0
Elevation	-0.483	0.096	-5.039	0
Elevation^2	-0.082	0.085	-0.973	0.347
Random effects variance				
Site	0.11024			
Residual	0.02978			
Marginal R^2	0.634	Conditional R^2	0.922	
(e) Bacterial mineral				
	Estimate	Std. Error	t value	p-value
Intercept	8.839	0.081	109.093	0
Elevation	-0.265	0.057	-4.656	0
Elevation^2	-0.15	0.05	-2.976	0.01
Random effects variance				
Site	0.03779			
Residual	0.01341			
Marginal R^2	0.586	Conditional R^2	0.891	

Table S3. Full models of effects of climatic and edaphic parameters on α-diversity. Full models with all variables, showing a linear model for plants (n = 13) and linear mixed models for fungi/bacteria (with site as random effect; n = 42 (Final models with only significant variables are shown in Table S2). See Table 1 legend for definition of marginal/conditional R^2 for mixed models.

(a) Plants				
	Parameter	SE	t	p-value
Intercept	1.782	0.835	2.133	0.077
MAT	0.072	0.021	3.486	0.013
MAP	2.005e-04	4.495e-05	4.46	0.004
pH	0.186	0.218	0.851	0.427
totalC	0	0	-0.235	0.822
ECEC	-0.013	0.007	-1.762	0.129
resinP	-0.002	0.001	-1.776	0.126
Distance	-0.006	0.004	-1.248	0.240
R^2	0.977			
(b) Fungal organic				
	Parameter	SE	t	p-value
(Intercept)	5.88	0.826	7.115	0
MAT	0.002	0.014	0.12	0.906
MAP	-7.643e-05	4.858e-05	-1.573	0.138
pH	0.052	0.144	0.361	0.723
totalC	9.163e-07	7.336e-07	1.249	0.232
ECEC	-1.988e-02	4.909e-03	-4.049	0.001
resinP	0.001	0.001	1.331	0.204
Distance	0.0014	0.003	0.431	ns
Random effects variance				
Site	0.017539			
Residual	0.066038			
Marginal R^2	0.518745	Conditional R^2	0. 619739	
(c) Fungal mineral				
	Parameter	SE	t	p-value
(Intercept)	3.323	1.85	1.797	0.089
MAT	0.045	0.02	2.215	0.04
MAP	-8.233e-06	5.744e-05	-0.143	0.888
pH	0.385	0.406	0.95	0.355
totalC	0	0	0.038	0.97
ECEC	-0.012	0.009	-1.403	0.173
resinP	0.006	0.012	0.47	0.645
Distance	0.004	0.002	1.578	ns
Random effects variance				
Site	0.031851			
Residual	0.047036			
Marginal R^2	0. 411429	Conditional R^2	0. 649069	
Bacterial organic				
	Parameter	SE	t	p-value
(Intercept)	5.754	0.625	9.202	0
MAT	0.043	0.01	4.128	0.001
MAP	1.482e-04	3.676e-05	4.03	0.001
pH	0.444	0.109	4.088	0.001
totalC	-6.385e-07	5.552e-07	-1.15	0.269
ECEC	0.008	0.004	2.242	0.042
resinP	-0.003	0	-6.738	0
Distance	-0.0006	0.004	-0.1676	ns
Random effects variance				
Site	0.012723			
Residual	0.029783			
Marginal R^2	0. 8895862	Conditional R^2	0. 922635	
(a) Bacterial mineral				
	Parameter	SE	t	p-value
(Intercept)	6.85	1.801	3.803	0.001
MAT	0.031	0.02	1.579	0.13
MAP	1.097e-04	6.359e-05	1.725	0.108
pH	0.226	0.399	0.566	0.578
totalC	1.663e-06	2.430e-06	0.684	0.502
ECEC	0.003	0.008	0.441	0.662
resinP	-0.001	0.013	-0.102	0.92
Distance	0.003	0.0023	1.365	ns
Random effects variance				
Site	0.053670			
Residual	0.013623			
Marginal R^2	0. 4255028	Conditional R^2	0. 883695	

Table S4. Pairwise correlations between the measures of α-diversity in each of the five groups. Corresponding *p*-values are in parentheses; bold values indicate $p < 0.05$.

	Plants	Fungi Organic	Fungi Mineral	Bacteria Organic	Bacteria Mineral
Plants	1	0.390 (0.188)	**0.598 (0.031)**	**0.830 (<0.001)**	**0.565 (0.044)**
Fungi Organic	0.390 (0.188)	1	0.384 (0.175)	0.261 (0.367)	0.030 (0.92)
Fungi Mineral	**0.598 (0.031)**	0.384 (0.175)	1	0.394 (0.163)	0.451 (0.106)
Bacteria Organic	**0.830 (<0.001)**	0.261 (0.367)	0.394 (0.163)	1	**0.812 (<0.001)**
Bacteria Mineral	**0.565 (0.044)**	0.030 (0.92)	0.451 (0.106)	**0.812 (<0.001)**	1

Table S5. Elevational correlations in relative abundance of taxa. Values are Spearman correlation coefficients and are highlighted where ρ > 0.50 and $p < 0.05$.

Bacteria	Organic	mineral	Fungi	organic	mineral
Archaea;p_Crenarchaeota	**-0.47**	0.06	Ascomycota;c_*Archaeorhizomycetes*	**0.65**	**0.77**
Archaea;p_Euryarchaeota	0.22	**0.42**	Ascomycota;c_*Dothideomycetes*	**-0.65**	**-0.75**
Acidobacteria	0.23	**0.79**	Ascomycota;c_*Eurotiomycetes*	**-0.70**	**-0.74**
Actinobacteria	**-0.63**	**-0.70**	Ascomycota;c_*Lecanoromycetes*	0.00	-0.17
Bacteroidetes	**-0.45**	**0.65**	Ascomycota;c_*Leotiomycetes*	**0.64**	**0.64**
Chloroflexi	**-0.63**	**-0.72**	Ascomycota;c_*Pezizomycetes*	0.33	0.00
Elusimicrobia	**-0.50**	0.12	Ascomycota;c_*Saccharomycetes*	-0.03	0.00
Firmicutes	**-0.52**	**-0.58**	Ascomycota;c_*Schizosaccharomycetes*	0.00	-0.17
Gemmatimonadetes	**-0.59**	**-0.60**	Ascomycota;c_*Sordariomycetes*	**-0.61**	**-0.67**
Nitrospirae	**-0.50**	**-0.52**	Ascomycota; Other	0.06	0.32
Planctomycetes	0.17	-0.26	*Basidiomycota;c_Agaricomycetes*	0.05	-0.27
Proteobacteria;c_*Alphaproteobacteria*	**-0.59**	**-0.79**	Basidiomycota;c_*Microbotryomycetes*	**0.44**	**0.59**
Proteobacteria;c_*Betaproteobacteria*	**0.54**	0.17	Basidiomycota;c_*Pucciniomycetes*	0.00	-0.03
Proteobacteria;c_*Deltaproteobacteria*	**-0.53**	**-0.46**	Basidiomycota;c_*Tremellomycetes*	0.36	**-0.53**
Proteobacteria;c_*Gammaproteobacteria*	0.20	0.10	Basidiomycota; Other	-0.17	**-0.42**
Proteobacteria; Other	-0.01	0.22	Blastocladiomycota	0.00	0.00
Verrucomicrobia	-0.03	0.38	Chytridiomycota;c_*Chytridiomycetes*	-0.07	-0.05
Other	-0.21	0.27	*Chytridiomycota;Other*	**0.47**	0.32
			Glomeromycota;c_Glomeromycetes;o_Archaeosporales	-0.08	0.22
			Glomeromycota;c_Glomeromycetes;o_Diversisporales	**-0.50**	-0.17
			Glomeromycota;c_Glomeromycetes;o_Glomerales	**-0.50**	**-0.50**
			Glomeromycota;c_Glomeromycetes; Other	**-0.50**	0.00
			Other	-0.22	-0.01
